# Gene expression and metabolism preceding soft scald, a chilling injury of ‘Honeycrisp’ apple fruit

**DOI:** 10.1186/s12864-016-3019-1

**Published:** 2016-10-12

**Authors:** Rachel S. Leisso, Nigel E. Gapper, James P. Mattheis, Nathanael L. Sullivan, Christopher B. Watkins, James J. Giovannoni, Robert J. Schaffer, Jason W. Johnston, Ines Hanrahan, Maarten L. A. T. M. Hertog, Bart M. Nicolaï, David R. Rudell

**Affiliations:** 1 United States Department of Agriculture, Agricultural Research Service, Tree Fruit Research Laboratory, Wenatchee, WA USA; 2 School of Plant Science, Horticulture Section, Cornell University, Ithaca, NY14853 USA; 3 AgroFresh Solutions Inc. 130 Technology Center Way Wenatchee, Wenatchee, WA 98801 WA USA; 4 Boyce Thompson Institute for Plant Research, Cornell University, Ithaca, NY14853 USA; 5 United States Department of Agriculture, Agricultural Research Service, Plant, Soil, and Nutrition Laboratory, Ithaca, NY14853 USA; 6 The New Zealand Institute for Plant and Food Research, Ltd, Auckland, New Zealand; 7 The New Zealand Institute for Plant and Food Research, Ltd, Havelock North, New Zealand; 8 Washington Tree Fruit Research Commission, Wenatchee, WA USA; 9 BIOSYST-MeBioS, KU Leuven, Heverlee, Belgium

**Keywords:** *Malus* x *domestica* Borkh, Apples, Transcriptomics, Metabolomics, Chilling injury, Soft scald

## Abstract

**Background:**

‘Honeycrisp’ is an apple cultivar that is susceptible to soft scald, a chilling injury expressed as necrotic patches on the peel. Improved understanding of metabolism associated with the disorder would improve our understanding of soft scald and contribute to developing more effective management strategies for apple storage. It was expected that specific gene expression and specific metabolite levels in the peel would be linked with soft scald risk at harvest and/or specific time points during cold storage.

**Results:**

Fruit from nine ‘Honeycrisp’ apple orchards that would eventually develop different incidences of soft scald between 4 and 8 weeks of cold air storage were used to contrast and determine differential transcriptomic and metabolomic changes during storage. Untargeted metabolic profiling revealed changes in a number of distinct pathways preceding and concurrent with soft scald symptom development, including elevated γ-aminobutryic acid (GABA), 1-hexanol, acylated steryl glycosides, and free *p*-coumaryl acyl esters. At harvest, levels of sesquiterpenoid and triterpenoid acyl esters were relatively higher in peel of fruit that did not later develop the disorder. RNA-seq driven gene expression profiling highlighted possible involvement of genes and associated metabolic processes with soft scald development. These included elevated expression of genes involved in lipid peroxidation and phenolic metabolism in fruit with soft scald, and isoprenoid/brassinosteroid metabolism in fruit that did not develop soft scald. Expression of other stress-related genes in fruit that developed soft scald included chlorophyll catabolism, cell wall loosening, and lipid transport while superoxide dismutases were up-regulated in fruit that did not develop the disorder.

**Conclusions:**

This study delineates the sequential transcriptomic and metabolomic changes preceding soft scald symptom development. Changes were differential depending on susceptibility of fruit to the disorder and could be attributed to key stress related and mediating pathways.

**Electronic supplementary material:**

The online version of this article (doi:10.1186/s12864-016-3019-1) contains supplementary material, which is available to authorized users.

## Background

Cold storage can result in various necrotic injuries of the peel or flesh of apples that can render the product unmarketable. Soft scald of ‘Honeycrisp’ and other susceptible cultivars is caused by cold stress and is typically comprised of ribbon-like browned and sunken peel tissue, with sharply demarcated edges [1, 2]. Economic losses resulting from this disorder can be substantial, and incidence is unpredictable among years and even among orchards in the same region [3] and from tree-to-tree [4, 5]. Pre-harvest conditions influencing soft scald risk include orchard climate [4] and harvest date, where fruit from later harvests are more susceptible to soft scald [6]. Treatments reducing soft scald incidence include pre-harvest application of the ethylene-action inhibitor 1-methylcyclopropene (1-MCP) [7] and postharvest temperature conditioning, where fruit are acclimated at ~10 °C for 7–10 d prior to colder storage at ~3 °C [3, 8].

Pre- and postharvest fruit physiology associated with soft scald incidence has been difficult to determine. The success of treatments such as temperature conditioning prior to cold storage for alleviating soft scald, in addition to increased disorder incidence and severity at lower temperatures, indicate soft scald is a chilling injury [3]. Also, greater soft scald incidence on more mature fruit suggests that physiological processes related to ripening may result in enhanced disorder susceptibility [6]. 1-Hexanol production has also been linked with soft scald, as the compound accumulates in symptomatic fruit, and exogenous application can enhance disorder development [9].

Previous research has enabled progress towards utilizing metabolomic approaches to better understand apple postharvest fruit disorders as in the case of superficial scald, a disorder resulting in necrosis of patches of apple peel 5–6 cell layers deep after several months of storage [10]. Superficial scald is linked with increased levels of oxidized metabolites, including conjugated trienols which result from α-farnesene oxidation [11]. These metabolites occur prior to and alongside disorder development [12–14]. Levels of other compounds associated with a typical metabolic fingerprint, including multiple aroma volatiles, are diminished in symptomatic tissue [13]. Acylated sterol glycoside (ASG) accumulation alongside diminishing steryl ester content also occurs prior to superficial scald demonstrating coordinated membrane component transition associated with symptom development [15].

Other etiologically distinct chilling provoked disorders, including soft scald, remain less metabolically characterized than superficial scald. ‘Honeycrisp’ cortex tissue affected by soggy breakdown, an internal disorder often associated with soft scald, contains reduced levels of catechins, carotenoids, and triacylglycerides, while levels of ethanol and ethyl esters are elevated within the symptomatic tissue [16]. Other internal browning disorders, including internal CO_2_ injury, are also associated with fermentation and related volatile production [17, 18]. Elevated γ-aminobutryic acid (GABA) and glutamate concentrations are associated with multiple internal browning injuries including CO_2_ injury [18], firm flesh browning, a diffuse injury occurring months after storage imposition [19], and soggy breakdown [16].

Relatively few studies have utilized gene expression data combined with metabolic profiling to understand apple postharvest disorder development. A study using a RNA-seq approach, indicated that external CO_2_ injury, a peel disorder of ‘Empire’, may be related to the methylation state of specific promoter regions [20]. RNA-seq was used to evaluate transcriptomic changes preceding internal CO_2_ injury in ‘Jonagold’ indicating gene expression related to energy metabolism and ethylene regulation were most impacted in tissue expected to develop symptoms [21]. Another multi-genic study targeted catalytic genes involved in phenolic metabolism and the impacts of superficial scald symptom development, linking the disorder with polyphenol oxidase gene expression while expression of other genes involved in catalytic steps of phenolic metabolism remained unchanged [22].

The objective of this study was to determine specific metabolites and expressed genes which are unique to fruit peel at risk for developing soft scald during storage. We hypothesized that gene expression differences and differences of metabolite levels would exist at-harvest and following cold storage imposition among orchards depending upon soft scald risk. We expected that analysis of metabolic profiles and gene expression profiles would reveal biochemical pathways related to the inhibition or risk of disorder development.

## Results

### Fruit maturity, quality, and soft scald incidence

**Fig. 1:**
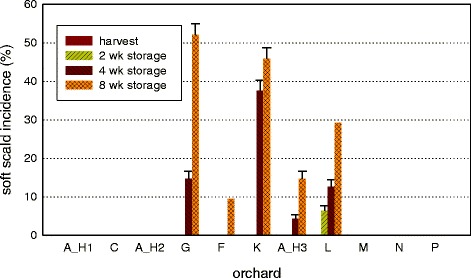
Soft scald incidence at 8 weeks of storage on fruit from Washington State orchards selected to provide the greatest contrasts of soft scald incidence and difference of geographic location. Orchards are listed in order of harvest date, which is available in Additional file 1: Table S1. A_H1, A_H2, and A_H3 are three sequential harvests from the same orchard. A_H2 had 6 % disorder incidence at 12 weeks and was therefore categorized as a susceptible orchard. Error bars represent standard error (*n* =3 replicates of 16 fruit)

Harvest maturity assessment indicated that apples used for metabolomic and transcriptomic analysis were harvested at different maturities among orchards (Additional file 1: Table S1). Soft scald incidence was also different depending upon the orchard where the fruit was grown (Fig. 1), and there was no clear association between harvest maturity, harvest date, and soft scald risk. Later sequential harvests from orchard A had higher soft scald incidence than earlier harvests, but fruit from later harvest dates from other locations did not have higher disorder incidence. Expression of genes upregulated with apple fruit ripening did not indicate clear differences of maturity/ripeness at harvest, during storage, or associated with soft scald risk (Additional file 2: Figure S1).

### Untargeted multivariate analysis of gene expression and metabolism

RNA-seq was used to evaluate the transcriptomic changes in nine orchards at harvest. Three of these orchards (orchards A, M, and P) also had samples analyzed at 2, 4, and 8 weeks; furthermore, fruit from orchard A were harvested 3 times at 2 week intervals (H1, H2, and H3). An average of 4.37 million reads were produced per sample, and 84 % mapped to the apple genome [23]. 53,411 genes were consistently detected in all samples out of the 63,523 physically annotated genes. The dataset was reduced to ~23,400 genes where expression exceeded an average value of 4 RPKM and, then, to the 5000 gene models that had the greatest variability among the conditions employed as experimental contrast. These were used to generate the principal components analysis (PCA) and ANOVA simultaneous components analysis (ASCA) models. Over 800 metabolites were used to generate the PCA and ASCA models for the metabolomic evaluation.

**Fig. 2:**
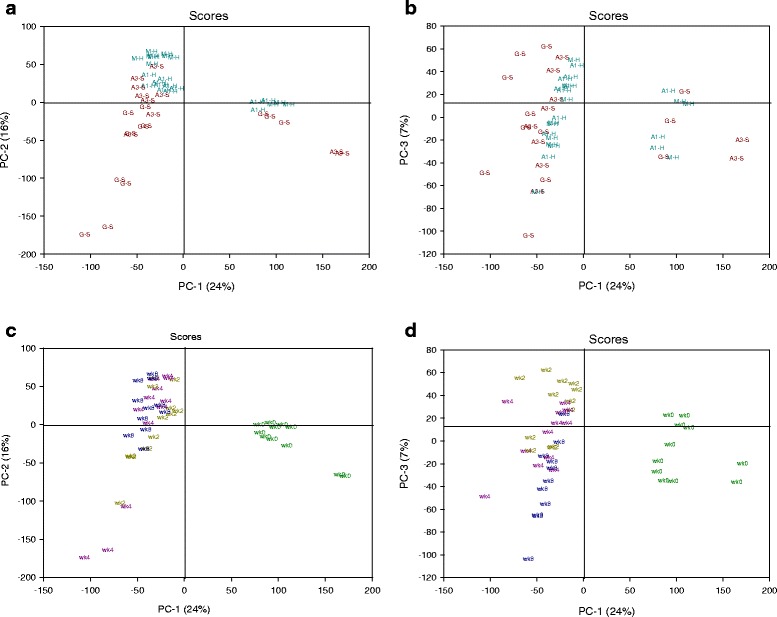
Principal components analysis (PCA) of ‘Honeycrisp’ apple peel transcriptomic data sampled from 0 to 8 weeks from multiple orchards and stored in air at 1 °C for up to 8 weeks. Color coding on the scores plot illustrates: **a**–**b** Differences between fruit peel samples taken from orchards that developed (High risk; S) or were free of soft scald (Low risk; H) in the first three principal components and **c**–**d** separation among fruit peel samples taken at harvest (wk0) and postharvest storage samples taken at 2, 4, and 8 weeks (wk2–wk8) in the first three principal components

**Fig. 3:**
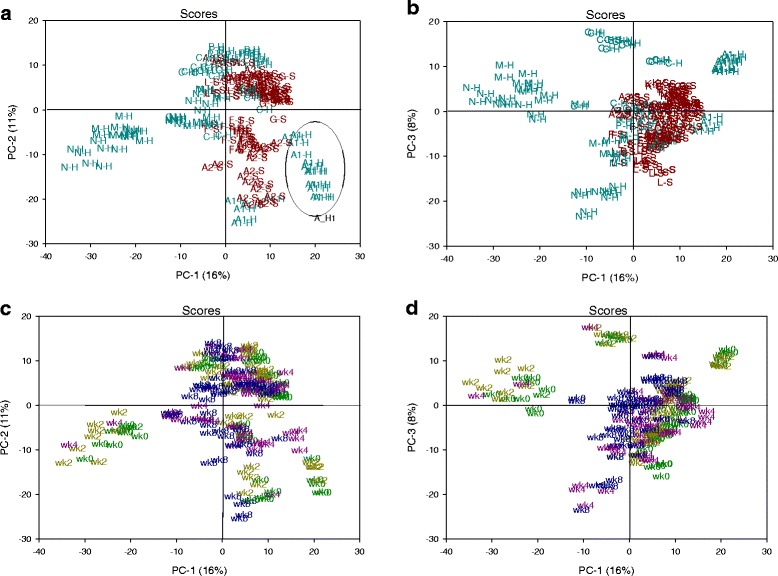
Principal components analysis (PCA) scores plots of metabolomic data from ‘Honeycrisp’ apple peel sampled from 0 to 8 weeks from fruit harvested from multiple orchards and stored in air at 1 °C for up to 8 weeks. The first letter in plots **a** and **b** indicate the orchard. In plot A, color coding illustrates general divergence between scores in the first two principal components (PCs) from fruit peel samples taken from orchards that developed (High risk; S) or were free of soft scald (Low risk; H), with the exception of A_H1 orchard (scores circled). **c** and **d** Color coding and labelling indicate differences associated with postharvest storage duration (wk0–wk8)

PCA was used to compare the transcriptome and metabolome among orchards during the 8 week storage period. Separate models were generated for each global dataset since the metabolome evaluation included more orchards. The transcriptome scores indicated substantial change between harvest and later storage (Fig. 2c and d, scores coded by storage week) with transcriptomic divergence between fruit with low risk and high risk for soft scald (Fig. 2a and b, scores coded by soft scald susceptibility) in the first three principal components. Fruit from low risk orchards tended to have lower PC2 scores at all sampling points than those from high risk orchards (Fig. 2c). A relatively small portion of the overall variance was accounted for by the first three principal components of this model (PC1, 24 %; PC2, 16 %; PC3, 7 %). The relative divergence between at-harvest and 2 week scores indicated the initiation of cold storage accounted from much of the variance in PC1-2. Following the first 2 weeks of storage, scores were not different between high risk and low risk fruit, but demonstrated the continued impact of storage duration on the transcriptome by separation of 8 wk scores in PC3 (Fig. 2d). The PCA model generated using the metabolome indicated differences among samples according to soft scald risk in the first two principal components (Fig. 3a), with the exception of peel from the first harvest of orchard A, which were less mature at harvest (Additional file 1: Table S1). The metabolome of orchard N from 4 to 8 week was different than the others in this plane (Fig. 3c and d). As with the transcriptome model, a relatively small portion of the total variance contributed to the first three principal components (PC1, 16 %; PC2, 11 %, PC3, 8 %).

### Metabolomic and transcriptomic divergence related to soft scald development in storage

**Fig. 4:**
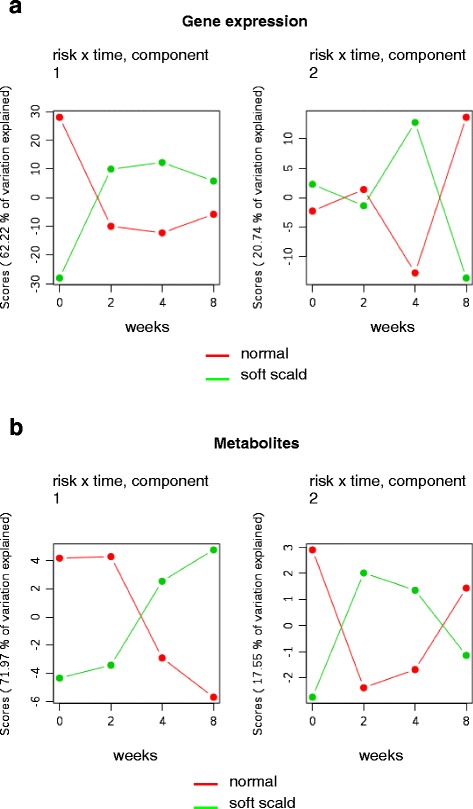
The first two components (ASCA) indicating the interaction of soft scald risk and storage time (weeks) for gene expression (**a**) and metabolites (**b**) in ‘Honeycrisp’ apple fruit

ASCA was used to reveal transcripts and metabolites which were significantly up or down regulated over the storage period in each risk category. ASCA models of the metabolomic and transcriptomic datasets also illustrated the transition of the transcriptome starting at the beginning of cold storage while revealing key differences in the metabolome within each soft scald risk category that were not clear in the PCA model. The ASCA model supported the PCA model with respect to the widespread transcriptomic change provoked by cold storage. When plotted over the storage period, the first two ASCA components (62 % of the variance) summarized transcriptomic changes in response to chilling, with scores of fruit from high risk orchards increasing and low risk decreasing (Fig. 4a). While still explaining 21 % of the variance in the model, changes in the scores of the second component over time were not clearly associated with any obvious experimental factor.

Component one (72 % of the variance) of ASCA model compiled using the metabolome revealed scores that decreased in low risk fruit and increase in high risk fruit between 2 and 4 weeks, or around the time when the first soft scald symptoms begin to appear (Fig. 4b). Taken together, the ASCA models of the transcriptome and metabolome illustrated an immediate response to the cold storage by the transcriptome followed by the metabolome beginning to take place around the time of symptom development in fruit peel from high risk orchards.

**Fig. 5:**
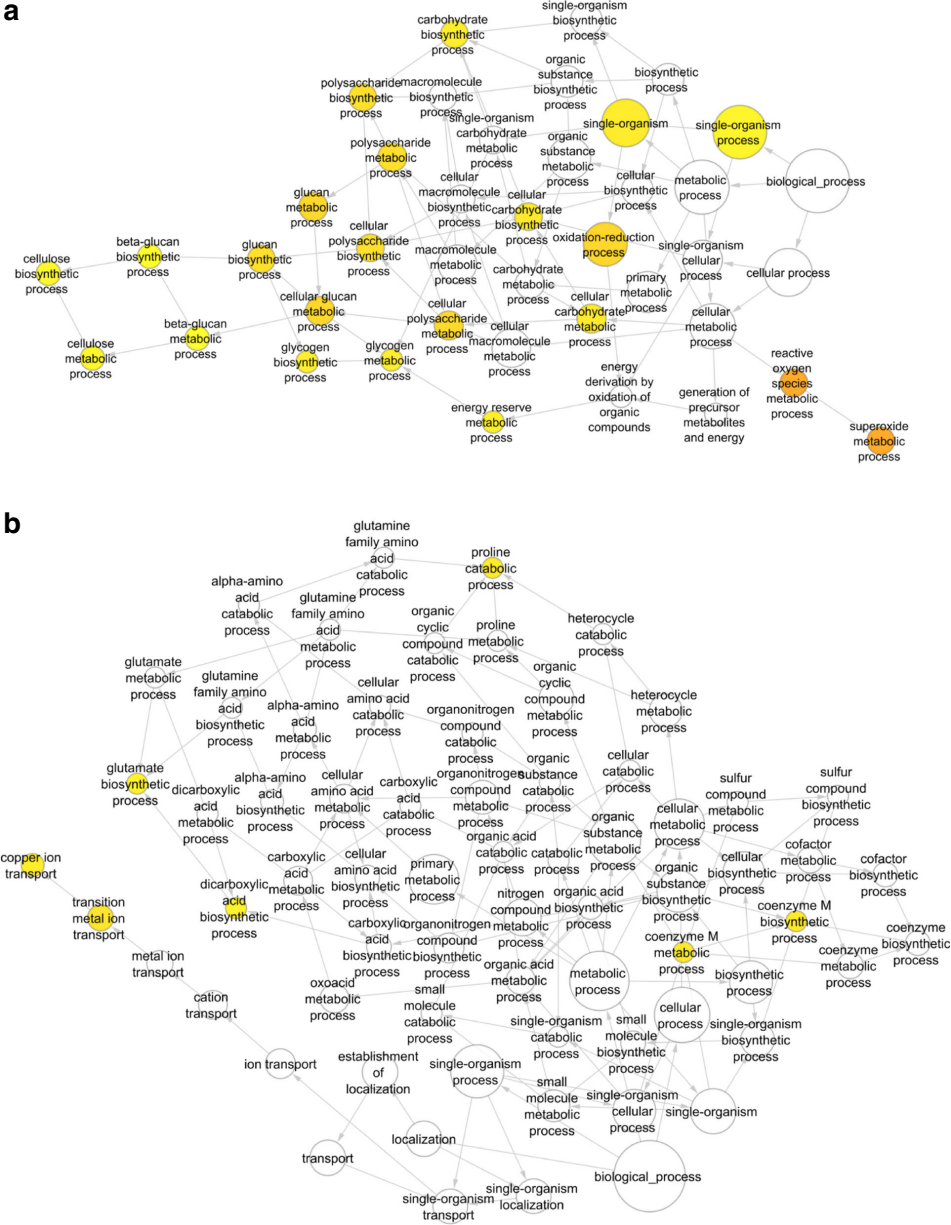
BiNGO overexpression analysis (processes) of significantly up-regulated gene models over the 8 week storage period from fruit harvest from low risk (**a**) and high risk (**b**) orchards in the ASCA “risk*time” model. Colored nodes represent GO terms significantly overrepresented; the intensity of *orange color* indicates *p*-value and size represents the number of genes

BiNGO overexpression analysis and visualization was chosen to generate GO networks highlighting biological processes that were differentially up-regulated, as determined by ASCA analysis, during storage for fruit from high risk and low risk orchards. Processes that were differentially up-regulated during storage of apples that did not later develop soft scald (Fig. 5a) are largely related to energy production including carbohydrate and polysaccharide biosynthesis. Superoxide and other reactive oxygen species metabolic processes were up-regulated in these fruit as was expression of multiple superoxide dismutase (SOD) genes indicating these fruit mustered a coordinated response to stress beginning at 4 weeks but not earlier given there were no differences in these processes in low risk fruit at-harvest or at 2 weeks (see below). A number of SODs were, instead, more highly expressed at-harvest, but not beyond, in high risk fruit (see below). Up-regulated biological processes in high risk fruit (Fig. 5b) involved amino acid metabolism including glutamate biosynthesis, glutamine, and proline catabolism, specifically proline dehydrogenase activity. Other processes included copper ion transport and iron ion homeostasis processes as well as oxidoreductase, acetoacetate synthase, and iron binding activity.

**Fig. 6:**
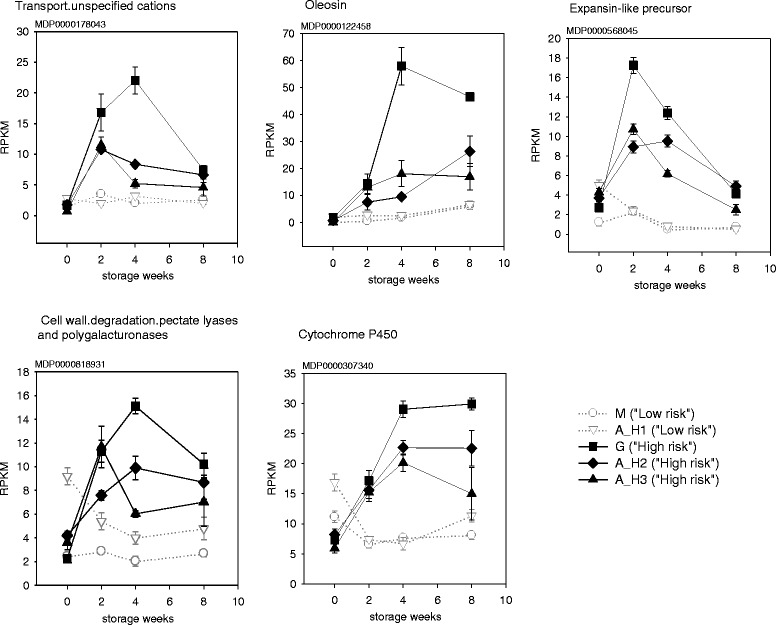
Genes that remained consistently up-regulated after cold storage imposition in fruit harvest from high risk orchards. Error bars represent standard error (*n* = 3)

Other genes consistently up-regulated over the entire storage period in fruit that developed soft scald included cation transport (MDP0000178043), an expansin-like precursor (MDP0000568045) and pectate lyase (MDP0000828931), a cytochrome P450 (MDP0000307340), and an oleosin (MDP0000122458) (Fig. 6). Among fruit that did not develop soft scald, the list included another cytochrome P450 (MDP0000692178), a deoxyxylulose-5-phosphate synthase (MDP0000253952) and two squalene epoxidases (MDP0000202883 and MDP0000638870), all of which can be involved in isoprenoid metabolism.

### Soft scald risk transcriptomics and metabolomics preceding disorder development

RobiNA was employed to analyze differential gene expression of fruit from all orchards at-harvest according to soft scald risk, and differential gene expression at 2 weeks of fruit from three orchards according to soft scald risk, including fruit from an orchard with three sequential harvest dates (each harvest treated as an individual orchard). As the PCA and ASCA analysis indicated, most of the change in the transcriptome occurred during cold storage and it was expected that expression of genes associated with high or low risk fruit at-harvest or 2 weeks may not remain different across the whole storage period. Consequently, to account for changes occurring during the first 2 weeks of storage, transcriptomic and metabolomic data were individually compared at each of these time points.

**Fig. 7:**
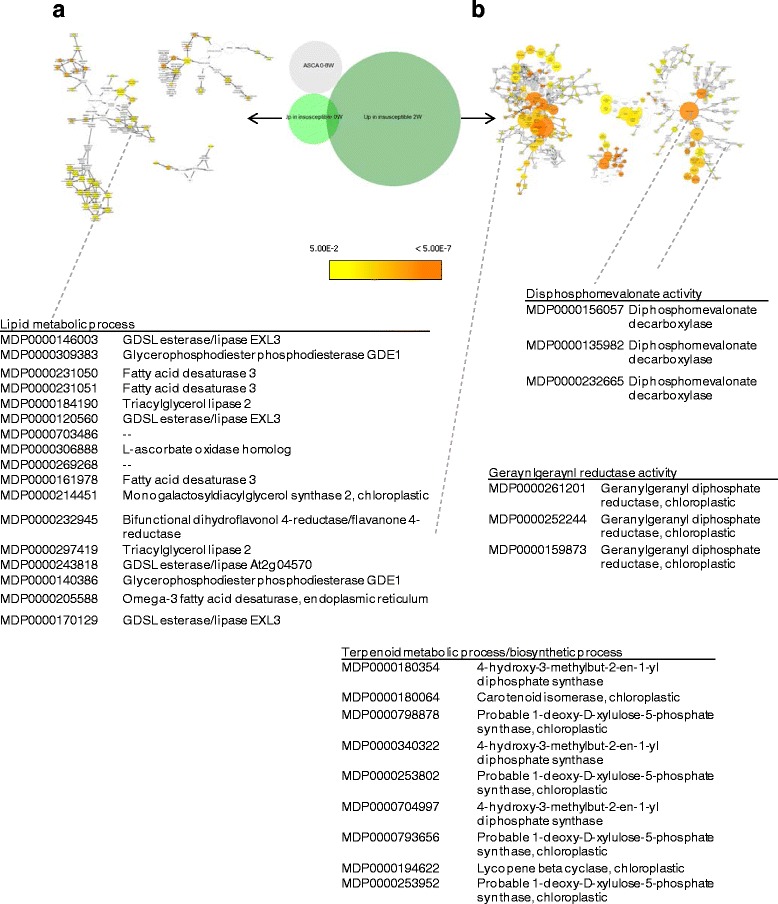
Overrepresentation analysis of genes elevated in fruit from orchards with low risk for soft scald at harvest (**a**) and 2 weeks (**b**). Colored nodes represent GO terms significantly overrepresented; the intensity of orange color indicates *p*-value and size represents the number of genes included in the category

**Fig. 8:**
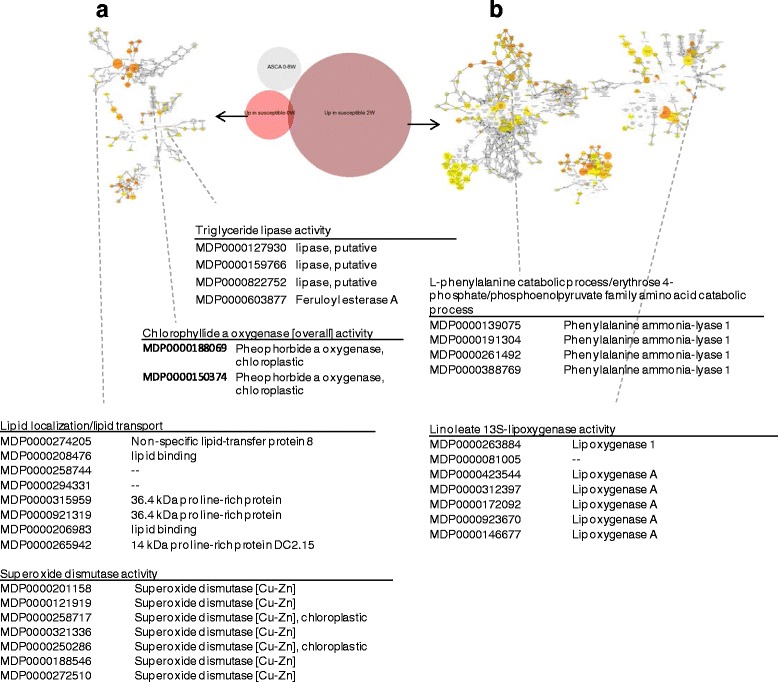
Overrepresentation analysis of genes with levels elevated in fruit from orchards with high risk for soft scald at harvest (**a**) and 2 weeks (**b**). Colored nodes represent GO terms significantly overrepresented; the intensity of *orange color* indicates *p*-value and size represents the number of genes

Eight hundred fifty-five genes were differentially expressed at harvest and 5726 genes were differentially expressed at 2 weeks. There were differentially expressed genes (DEG) in common for both the at-harvest and 2 weeks lists from fruit from both the low risk and high risk orchards (Additional file 3: Figure S2). Multiple GO process categories of at-harvest and 2 week DEGs were overrepresented in fruit from both low risk (Fig. 7) and high risk orchards (Fig. 8).

Up-regulated genes at-harvest in fruit from high risk orchards included multiple superoxide dismutases (MDP0000201158, MDP0000121919, MDP0000258717, MDP0000321336, MDP0000250286, MDP0000188546, MDP0000272510), triacylglyceride metabolism, and genes involved in stress-related chloroplastic metabolism. At 2 weeks following cold storage imposition, multiple lipoxygenases (MDP000262884, MDP000081005, MDP0000423544, MDP0000312397, MDP0000172092, MDP0000923670, MDP0000146677) and phenolic metabolism related genes (MDP0000139075, MDP0000191304, MDP0000261492, and MDP000388769), both classes indicative of a stress response, were also up-regulated.

Up-regulated genes at-harvest in fruit from low risk orchards included genes involved in lipid metabolism. Following the beginning of cold storage, up-regulated genes included isoprenoid metabolism and, unlike those grown in high risk orchards, other metabolic processes ostensibly transpiring in the chloroplast. Triacylglyceride (TAG) lipase, superoxide metabolic process, and reactive oxygen species metabolic processes categories were overrepresented both low risk and high risk fruit. Although there were SODs categorized into this process, they were different genes depending upon the risk category. Overall, differential gene expression related to cold storage imposition of fruit from high risk orchards was stress-related, while gene expression was related to stress mediation and normal chloroplastic function in low risk fruit.

No genes were common between the DEG and the experiment-wide ASCA analyses for fruit from either low or high risk orchards, although some genes were up-regulated at-harvest and 2 weeks in both risk categories (Additional file 3: Figure S2). Up-regulated processes at both 0 and 2 weeks in fruit from low risk orchards included only two phytosulfokine genes which were categorized as “growth factor activity” and “cell proliferation”. In fruit from high risk orchards, these included transferase activity, specifically C6, redox activity, ion binding, and transcription factor binding. Interestingly, RNA binding factors included in this list were entirely Myb transcription factors.

Metabolites from multiple biochemical pathways different between fruit from high and low risk orchards according to risk and risk × time over the entire storage period are listed in Table 1. GABA levels were higher in fruit from high risk orchards over the whole storage period. Other metabolites, also elevated with risk, included a monogalactosyldiacylglyceride (MGDG; C18:2, C18:2), 1-hexanol, 2-methylbutanol, β-alanine, and two partially identified *p*-coumaryl acyl esters. Apple peel from low risk orchards contained elevated levels of multiple classes of metabolites across the storage period. Among these were multiple isoprenoid compounds including farnesyl oleate, farnesyl linoleate, and farnesyl linolenate as well as one other partially identified farnesyl acyl ester. In addition to these, three partially identified triacylglycerides (TAGs) were elevated in this tissue (Table 1). Shikimic and aspartic acid content were also higher in this risk category.

**Table 1:** Identified, partially identified, and unidentified metabolites different between risk categories in ‘Honeycrisp’ peel from orchards at low or high risk to develop soft scald by 8 weeks cold air storage overall (ASCA; leverage = 0.90 and alpha = 0.001). Abbreviations and mass spectral tags are included in columns 2 and 3, respectively. The evaluation method usedfor each component were non-polar (no symbol), volatile (*), or TMS-Oxime (**) (see second column)^a^

ID or tentative ID	Metabolite	Mass spectral tag (RT, *m/z* Target ion)
		*low risk orchards*
	trienol735	(18.8, 203.1789)
	LC276	(21.5, 109.1024)
	LC154	(17.2, 556.5310)
	LC641	(11, 335.2938)
	LC27	(24, 919.7877)
	LC203	(24.3, 381.3709)
butyl 2-methylbutenoate^c^	But2MButen	(7.4, 57)*
	sester167	(18.1, 203.1809)
	LC421	(23.4, 365.342)
	LC196	(23.1, 367.3574)
	LC278	(24.7, 395.3835)
	chlor660	(12.4, 337.3098)
	LC634	(13.3, 283.2654)
	sester642	(18.1, 205.1952)
	LC201	(23.7, 391.3569)
	LC414	(23, 391.3581)
	LC156	(15.7, 367.3565)
	LC210	(15.1, 365.3413)
	LC403	(22.4, 391.3575)
TAG(18:3,18:3,18:3)^g^	TAG420	(23.3, 873.6960)
	LC657	(11.8, 207.1737)
	LC589 TAG	(23.3, 595.4729)
Z,E α-farnesene^c^	Zefarn	(12.8, 93)*
	LC194	(22.8, 365.3414)
	LC198	(23.4, 389.3412)
	LC557	(14.8, 631.4927)
	LC531	(14.5, 366.3725)
	sester650	(11.6, 205.1945)
	LC408	(22.6, 389.3434)
	LC171	(18.6, 505.4271)
	sterB481	(7.8, 441.3738)
	LC204	(24.2, 393.3711)
	LC783	(33.3, 681.6336)
aspartic acid^c^	Asp	(10.5, 232.2)**
	LC623	(24.5, 573.4877)
	LC358	(19.7, 565.5668)
	LC775	(27.1, 575.5033)
	LC356	(19.5, 335.2596)
TAG(18:1,18:1,18:2)^g^	TAG226	(27, 601.5177)
	urs474	(7, 423.3257)
	LC386	(21.2, 341.3054)
	LC192	(22, 341.3065)
Farnesyl oleate^e^	sestc389	(21.3, 205.1647)
	LC341	(18.8, 665.55)
	LC28	(24.2, 945.7895)
	LC120	(26.3, 573.4893)
TAG(18:2,18:2,18:1)^g^	TAG442	(25.9, 601.5184)
Farnesyl linolenate^e^	sestc644	(19.5, 205.1943)
Farnesyl linoleate^e^	sestc546	(20.3, 205.1952)
	ster263	(3.14, 421.3251)
	LC354	(19.4, 599.5039)
	photo431	(24, 749.6224)
shikimic acid^c^	ShiA	(12.3, 204.1)**
	LC388TAG	(21.3, 603.5349)
		*high risk orchards*
2-methylbutanol^c^	2MButol	(3.7, 57.1)*
γ-aminobutyric acid^c^	GABA	(10.6, 304.2)**
β-alanine^c^	B-Ala	(9.8, 248.2)**
	LC620	(22.1, 631.5646)
	LC598	(8.18, 379.3173)
1-hexanol^c^	1-Hex	(56, 5.2)*
	LC583	(21.4, 629.5497)
	LC643	(18.9, 165.0918)
glutamic acid^c^	GluA	(11.1, 246.2)**
	GCT204	(24.2, 393.3711)
	LC558	(15.3, 615.4986)
	LC149	(14.1, 633.5057)
	LC596	(6.8, 365.3060)
*p*-coumaryl ester^f^	comacyl659	(12.2, 133.0653)
acetic acid^c^	Ace	(3.1, 60)*
	LC202	(23.8, 367.3585)
	LC636	(14, 309.2795)
MGDG (18:2,18:2)^b^	MGDG313	(17.7, 617.5127)
1-propanol^c^	1-Pro	(2.4, 31)**
*p*-coumaryl ester^f^	comacyl523	(13.4, 133.0645)
	LC4	(2.3, 219.1729)
	LC19	(19.7, 893.5409)
	LC390	(21.4, 395.3677)
campesteryl glucosyl linoleate^d^	CGL	(21.9, 383.3662)
	LC309	(17.6, 577.4825)
	LC180	(20.2, 669.5805)
2-ethylhexanol^c^	2EtHexol	(7.5, 57.1)*

^a^See text for method and instrumental specifics

^b^Standard from Avanti Polar Lipids, Inc, Alabaster, Alabama

^c^Standard from Sigma-Aldrich, St. Louis, Missouri

^d^See Rudell et al., 2011. Phytochem. 72:1328-1340

^e^See Additional file 7: Protocol S1

^f^Standard acquired for *p*-coumaryl stearate and partial identification of other *p*-coumaryl esters from Bruce D. Whitaker. See Whitaker, B.D. 2001. J. Agric. Food Chem. 49:3787-3792

^g^Partially identified using mass spectrum and accurate mass. Exact position of acyl groups is unknown

**Fig. 9:**
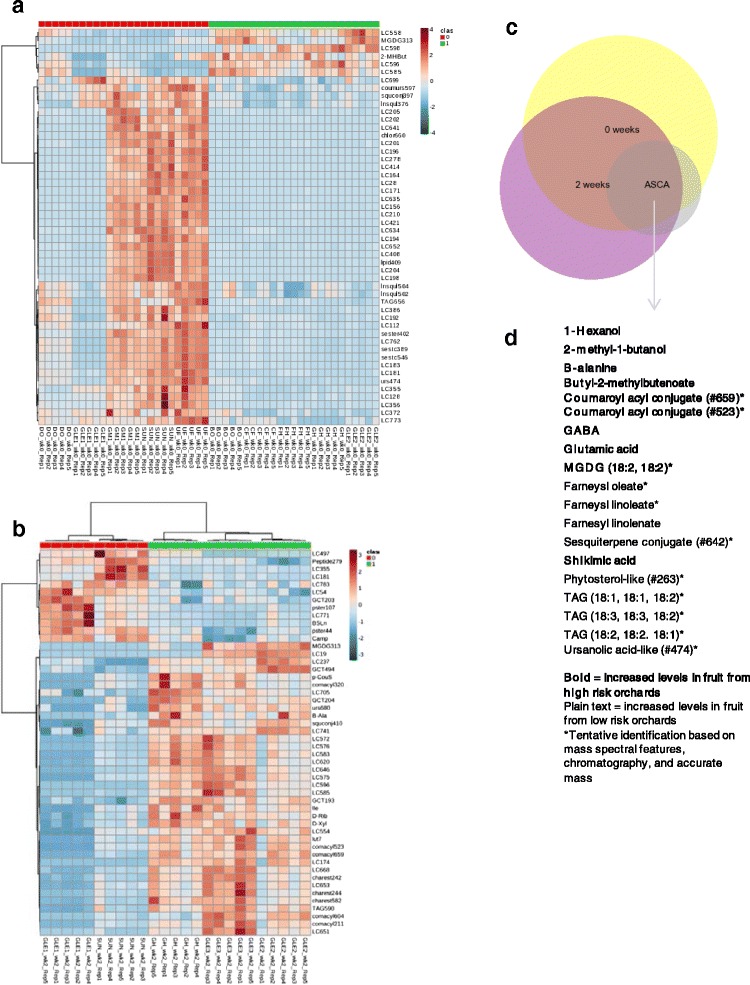
The top 50 metabolites differentiating orchard soft scald risk at harvest (**a**) and after 2 weeks of cold storage (**b**), organized using hierarchical cluster analysis. In total, 277 metabolites were significantly different at harvest, and 329 at 2 weeks, among low risk (*red*) and high risk (*green*) orchards. Levels of 65 of these metabolites were different at both time points (**c**), of which 19 are tentatively or partially identified (see Table 1) (**d**)

Metabolites were also directly compared between risk categories at-harvest and 2 weeks to indicate whether early changes in the metabolome could be linked with soft scald risk. As indicated by the summary of metabolomic change in the ASCA model (Fig. 4b), the impact of cold storage was not as immediately apparent on the metabolome as it was on the transcriptome. In all, 374 metabolites were significantly (α = 0.05) linked with risk category at-harvest and 329 at 2 weeks. The top 50 metabolites (in significance) at-harvest and 2 weeks were clustered using Pearson’s correlation coefficient and Ward’s clustering algorithm and represented as a heatmap (Fig. 9). Differences between soft scald risk categories at these early time points was less dramatic and, many times, less consistent than the transcriptome. This is clear where the first harvest of orchard A (OrchA_H1) was in the low risk category but grouped independently of other low risk orchards in the first principal components in an unsupervised multivariate analysis (PCA) (Fig. 3a). During cold storage, the differences in the both the metabolic and transcriptomic profile of orchard A, first harvest (Orch A_H1) were not as important for explaining the distribution of variance in these factors in the PCA model, and did not group separately in the first several principal components in PCA. The PCA model explained less of the variation than ASCA, and as such, should be interpreted in tandem with and in the context of other analyses. Fruit from low risk orchards had elevated levels of other isoprenoids at harvest. Levels of an unidentified steryl myristilate (21.7, 409.3824; [RT, m/z target ion]) were higher in peel from high risk orchards at harvest. Levels of two unidentified sterols (10.7, 409.4000; 11.5, 409.3788) were also higher in these samples but were the same in all orchards following cold storage imposition. Fruit from low risk orchards also had higher levels of two other unidentified steryl esters, a steryl laurate and a steryl palmitate (20.7, 409.3827; 22.7, 409.3841) at 2 weeks but not beyond. Comparison with authentic 2,3-epoxysqualene, cycloartenol, β-amyrin, and α-amyrin standards produced chromatographic peaks that indicated the unknowns are more polar as did the previous standards esterified with lauric and palmitic acid. In addition to elevated campesteryl (6’-O-linoleoyl) β -D-glucoside (CGL) content in fruit from high risk orchards throughout storage, other parts of phytosterol metabolism were also impacted by soft scald risk at-harvest or immediately following cold storage imposition. Campesterol and β-sitosteryl linoleate (BSL) levels were elevated at 2 weeks in fruit from high risk orchards.

Metabolites with significantly different levels at-harvest and/or 2 weeks and also in the ASCA model were considered the most accurate appraisal of metabolism associated with scald risk (Fig. 9). These included many of the aforementioned components, particularly GABA, glutamate, β-alanine, shikimic acid, MGDG, and tentatively identified *p*-coumaryl esters in high risk orchards. Levels of 3 TAGs as well as the farnesyl esters were consistently higher in fruit from low risk orchards at-harvest and for the entire storage period.

Comparison of gene expression and metabolite results indicated that gene expression both preceded or occurred alongside changes in levels of linked metabolites. However, links between gene expression associated with direct regulation or production of a metabolite and levels of that metabolite were not obvious. Other relationships between gene expression and metabolite levels did occur together exclusively in peel from high or low risk orchards and indicate, at the very least, possible links between genetic regulation and metabolite levels as impacted by scald risk. Examples of potential coordinated activity include elevated LOX expression following cold storage imposition preceding increased 1-hexanol levels in high risk orchards, genes involved in glutamic acid biosynthesis and glutamine metabolism were up-regulated alongside elevated GABA levels in fruit from high risk orchards, upregulation of PAL and *p*-coumaryl ester in fruit from high risk orchards, and genes related to isoprenoid metabolism with elevated levels of α-farnesene, farnesyl esters, unidentified sterols, and unidentified steryl esters in apples from low risk orchards.

Correlation analysis (Additional file 4: Figures S3 and Additional file 5: Figure S4) revealed a few highly correlated changes between gene expression and metabolites although none with obvious regulatory or mechanistic associations. In separate correlation networks comprised of 4387 expressed genes/ metabolites found in high risk fruit and 880 expressed genes /metabolites found in low risk fruit, levels of few metabolites and transcripts were highly correlated (Additional file 5: Figures S4 and Additional file 6: Table S2). A subnetwork containing highly correlated genes and metabolites (*r* ≥ 0.95) in high risk fruit contained Zinc finger proteins and a protein translation factor, (MDP0000170739, MDP0000224773, MDP0000232642, MDP0000606526) alongside acetaldehyde and hexyl 2-methylbutyrate. Subnetworks containing both upregulated genes and metabolites with higher levels in low risk fruit peel included three unidentified genes and a NADP-dependent D-sorbitol 6-phosphate dehydrogenase (MDP000503341, MDP0000209143, MDP0000429218, MDP0000873874, MDP0000133306) alongside succinic acid and an unidentified metabolite (1425.6, 160.1).

## Discussion

### Maturity and environmental effects on disorder incidence in relation to gene expression and metabolism

Previous studies have indicated fruit maturity impacts soft scald susceptibility, with later harvests typically having greater disorder incidence [6, 24]. In the present study, standard maturity indices (firmness, starch, Brix, titratable acidity) indicated differences of fruit maturity among orchards although advanced maturity was not always associated with soft scald risk (Table 1). Levels of other genes (Additional file 2: Figure S1) reported as upregulated in apples during ripening [25] further indicated the orchards used in this study represented a range of fruit maturities when harvested although not elevated soft scald risk in every case. There were other cases where transcript and metabolite levels associated with processes that may occur alongside advancing fruit maturity were also elevated in fruit from orchards with higher soft scald risk. For instance, a chlorophyllide a oxygenase (MDP0000188069, MDP000150374), an enzyme involved in chlorophyll degradation [26], was upregulated in fruit from high risk orchards at-harvest. Pheophorbide a oxygenase can increase via *de novo* synthesis both during senescence [27] and is proposed to be involved in chlorophyll catabolism during fruit de-greening [28], which occurs in apple peel during ripening [29].

These results indicate that, while maturity is important, other factors related to orchard and other pre-harvest conditions may have equal or greater impact on scald risk aside from maturity. Crop load [8] and environmental factors, such as orchard location and pre-harvest temperature [4], may influence gene expression levels and metabolic changes associated with soft scald risk. Incorporating these lat ent factors by using fruit from multiple orchards from relatively diverse locales was a critical to this evaluation. Metabolic differences among orchards were apparent in both the metabolomic and transcriptomic PCA models, which may result, in part, from the interaction of environmental conditions and fruit maturity. For example, fruit from orchard N, where metabolites clustered independently from other samples at 4 and 8 weeks, were obtained from an orchard 400 km from any other orchard with a similar scald outcome.

### Summary of overall transcriptomic and metabolomic changes

Results of both the PCA and ASCA illustrate an overall shift in the transcriptome between 0 and 2 weeks, or with the beginning of cold storage, followed by a shift in the metabolome between 2 and 4 week (Figs. 3 and 4). These trends outline a metabolic sequence where the transcriptome changes rapidly in response to chilling and, then, the metabolome as a consequence. Chilling can result in numerous cellular perturbations in plants, including lipid phase change and membrane leakage [30]. ‘Honeycrisp’ is sensitive to postharvest storage temperature as warmer storage temperatures are less likely to provoke soft scald, so changes beginning at this point may include various adaptive responses [31]. In this way, interrogation of the transcriptome and metabolome was a means for revealing which metabolomic and transcriptomic changes were most associated with symptom development.

### Metabolism differentiating soft scald risk categories

Different statistical analyses were aimed at examining metabolism in response to chilling as well as those over the entire storage evaluation. As the initial response to chilling seems to be staggered, it may not be expected that gene expression and the resulting metabolite products would be directly correlated during the initial response to cold storage. Because of this, any association between the two global data sets were based on similarities of GO processes and metabolite levels during this period. Nevertheless, pairwise correlation analysis did reveal a few highly correlate metabolites-gene combinations, albeit with no obvious regulatory or catalytic connection as annotated (Additional file 4: Figures S3 and Additional file 5: Figure S4).

### Glutamic acid and GABA metabolism in fruit from high risk orchards

Consistent gene expression in fruit peel from high risk orchards included processes involved in glutamate biosynthesis. Likewise, glutamic acid and GABA levels were elevated in fruit from high risk orchards at the time of first sampling from cold storage at 2 weeks and continuing through the final 8 week assessment. In a previous ‘Honeycrisp’ study, levels of GABA were also higher in tissue next to the browned tissue of soft scald/soggy breakdown [16]. GABA and alanine have also been reported to increase in response to low oxygen stress in ‘Granny Smith’ apples [32]. GABA may have a role in protecting plants from oxidative stress and its accumulation has been linked to abiotic stress in numerous other studies [33, 34]. β-alanine levels were also elevated in fruit from high risk orchards. β-alanine was also higher in the browned tissue of soggy breakdown, an internal storage disorder of ‘Honeycrisp’, in a previous study [16].

### Phenolic metabolism is impacted differentially depending on scald risk

At 2 weeks, five identified and partially identified *p*-coumaryl ester levels were elevated in fruit from high risk orchards. Two of these metabolites were significant in ASCA analysis, indicating levels continued to increase beyond initial cold stress. *p*-Coumaryl esters are major components apple peel cutin [35] and the presence of these monomers may represent unpolymerized substrate for cutin biosynthesis. Also, monolignols such as *p*-coumaryl alcohol are primarily precursors for ligin and lignan biosynthesis [36], the latter having activity in plant defense. In apple, studies have indicated these compounds increase in conjunction with light-induced anthocyanin accumulation and have antioxidant activity [37], and circumstantial evidence has attributed their presence to cultivar-specific resistance to superficial scald [38]. The *p*-coumaryl malate [39] and amide conjugates are also produced in response to pathogenic and abiotic stress [40].

Phenylalanine ammonium lyases (PAL) (MDP0000139075, MDP0000191304, MDP0000261492, and MDP000388769) were also overrepresented at 2 weeks in fruit from high risk orchards. PALs are involved in the first regulatory step of phenolic biosynthesis which includes the *p*-coumaryl esters as well as phenylpropanoids. Phenylpropanoids are a large class of chemical compounds that includes monolignols (precursors to lignin), flavanoids, proanthocyanidins, phenylpropanoid esters, and acylated polyamines [36]. In apple fruit tissue, PAL activity is enhanced by ethylene action during fruit ripening [41] and is associated with total flavanoid concentration [42]. In addition to some cutin monomers, *p*-coumaryl-CoA is the final precursor molecule preceding an array of phenylpropanoid compounds, including flavanoids, proanthocyanidins, phenylpropanoid esters, and acylated polyamines [36].

### Processes related to mediation of reactive oxygen species upregulated in low risk orchards with cold stress

As may be expected, metabolism associated with this period could be largely characterized as stress response, even in peel from low risk orchards. The longer-term storage transcriptomic trends (0–8 weeks) indicated reactive oxygen species (ROS) processes present in fruit peel from orchards that did not develop soft scald, including upregulated SOD genes. However, SOD was upregulated in fruit from high risk orchards prior to cold storage initiation. Previous apple postharvest disorder studies report that ROS are involved in postharvest disorder incidence or, possibly mediation [21], as ROS can have numerous adaptive relationships with stress [43].

### Elevated lipoxygenase expression and 1-hexanol content in fruit from high risk orchards

1-Hexanol content was higher in high-risk fruit at-harvest, 2 weeks, and overall during storage. Higher 1-hexanol levels have been detected prior to [44] and with soft scald symptoms [45]. 1-Hexanol, hexyl acetate, hexanal, and hexyl butyrate, applied to Jonathan apples, increased soft scald incidence [9]. As a result, it was hypothesized that hexanol is a toxic volatile that induces soft scald [44]. 1-Hexanol can be produced by β-oxidation of long-chain fatty acids [46, 47] but more likely via lipoxygenase activity [48]. Hydroperoxide lyase catalyzes hexanal biosynthesis from linoleic acid [48] which is, in turn, converted to 1-hexanol by alcohol dehydrogenase [49]. In ‘Anna’ apples, treatment with an ethylene action inhibitor (1-methylcyclopropene; 1-MCP) did not affect headspace 1-hexanol production [50], indicating that 1-hexanol production is not directly dependent on ethylene-mediated ripening processes, or possibly, not entirely associated with fruit ripening. In ‘Granny Smith’ apples, the concentration of esters containing the hexyl moiety increases during air storage in peel (16 weeks and 24 weeks) [51] which may indicate 1-hexanol production is related to senescence. In the current study, lipoxygenase (MDP000262884, MDP000081005, MDP0000423544, MDP0000312397, MDP0000172092, MDP0000923670, MDP0000146677) expression was overrepresented at 2 weeks of storage in peel from high risk orchards, although this result does not directly correspond with the elevated levels of 1-hexanol observed throughout storage. Other functions for lipoxygenases include defense signaling and peroxidation of membranes [52]. Their activity shortly after storage inception could result from the stress response to chilling temperatures. In summary, 1-hexanol levels may reflect an increase in fruit maturity at harvest, or the presence of pre-harvest stress.

### Sesquiterpenoid levels are enhanced in fruit from low risk orchards

Other non-polar compounds were elevated at harvest in fruit from low risk orchards including farnesyl esters as well as unidentified sterols and steryl esters. Farnesyl esters including farnesyl oleate, farnesyl linoleate, farnesyl linolenate and an uncharacterized sesquiterpene conjugate (18.1, 205.1952) were higher at-harvest and overall during storage in fruit from low risk orchards. These meroterpenoids are a type of natural compound containing both terpenoid and non-terpenoid components [53] and were recently first reported in higher plants as a component of ‘Gala’ apple wax [54]. Apple fruit produces both farnesol and α-farnesene which can be auto-oxidized to yield epoxides and endoperoxides and, then 2,6,10-trimethyldodeca-2,7(E),9(E),11-tetraen-6-ol [55]. The farnesyl esters with elevated levels in low risk orchards are esters of farnesen-15-ol and not the same as the α-farnesene oxidation product which was also detected, as were other partially identified farnesyl acyl esters that were not linked with any risk category. A physiological or metabolic role for these components in plants has not been established, although isoprenoid compounds may be an important component of membrane stability [56].

α-Farnesene is sesquiterpene produced by apple fruit. Studies suggest α-farnesene production occurs primarily in fruit peel [55], and early studies indicate it is the predominant sesquiterpene produced by apple [12]. Oxidation products of α-farnesene are associated with superficial scald of apples and pears [57]. In other organisms, sesquiterpene conjugates mitigate the response to nitric oxide [58] and sesquiterpene production increases in response to stress [59], although whether these sesquiterpenes have a similar role to those in the present study is unknown. In vitro, sesquiterpenes and sesquiterpene alcohols can reduce lipid oxidation [60]. The enzyme specifically involved with the synthesis of α-farnesene in apples (α-farnesene synthase [AFS1]) has been extensively evaluated [34, 61, 62] but, in the present study, levels of neither of the principal sesquiterpene metabolites in apple ([*E*,*E*] α-farnesene and [*Z*,*E*] α-farnesene) nor AFS1 expression were different based on scald risk.

Overexpression analysis supported the relationship between increased sesquiterpene synthesis and gene expression within this pathway. The GO category “Diphosphomevalonate activity” and deoxyxylulose-5-phosphate synthase expression were overrepresented in fruit from low risk orchards at 2 weeks. The former enzyme functions early in the mevalonic acid (MVA) pathway and precedes all isoprenoid biosynthesis in the cytosol/ER, including sesquiterpene and triterprenoid (phtyosterol) compounds [63]. The latter is the first and rate limiting step in chloroplastic isoprenoid biosynthesis [64]. Previous studies of other plant species indicate that sesquiterpene synthesis can take place via both the cytostolic MVA and chloroplastic methyl-d-erythritol 4-phosphate (MEP) pathways [65, 66], and even that prescursors specific to sesquiterpene synthesis can be transported between the chloroplast and cytoplasm [67], indicating potential modes of sesquiterpene synthesis previously unreported in apples.

### Phytosterol ester and triterpenoid biosynthesis

At 2 weeks, the identified phytosterols and phytosterol conjugates including β-sitosteryl linolenate (BSLn) and two unidentified metabolites with similar mass spectral peaks, were elevated in low risk fruit. Phytosterols are triterpenoid compounds synthesized via the mevalonic acid pathway located in the cytosol/ER [68]. An association between phytosterol composition and resistance to chilling injury in apple has been previously demonstrated, where steryl esters, such as BSLn, remained higher in fruit treated with antioxidant or ethylene action inhibitor to reduce superficial scald [15]. In ‘Honeycrisp’, another sitosteryl ester (β-sitosteryl linoleate), remained highest in the tissue of wholly healthy fruit, compared to tissue from fruit affected by soggy breakdown [16]. Also in ‘Honeycrisp’, acylated sitosteryl glucosides (palmitoyl and stearate) levels were higher in cortex of fruit with soggy breakdown following low temperature storage [16]. In the present study, neither whole fruit samples nor browned tissue were assessed separately at the time points when symptoms of disorders were present, so this phenomenon may not have been detectable. CGL levels were higher in high risk orchards throughout storage (ASCA), while campesterol was elevated in low risk orchards at 2 weeks. In tomatoes, chilling enhances levels of campesterol and isofucosterol [69], but increases of free sterols do not necessarily result in increases of conjugated sterols. Controls and mechanisms of phytosterol conjugation have only been sparingly characterized in plants and less so in fruit.

However, some evidence of upregulation of the initial steps in this pathway was observed. Levels of two squalene expoxidase (monooxygenase) (MDP0000202883 and MDP0000638870) transcripts were higher in fruit from low risk orchards throughout storage, although levels decreased in fruit from all orchards after 2 weeks. This enzyme catalyzes one of the early steps in sterol biosynthesis [70] and is thought to be rate limiting. Along with expression of the above genes, unidentified sterols, which appeared to be controlled by similar mechanisms as CTOL production with respect to superficial scald risk in ‘Granny Smith’ in our earlier work (unpublished) and two unidentified steryl esters (steryl laurate and steryl palmitate) levels, were higher in fruit from low risk orchards at harvest, although levels of an additional unidentified steryl esters were higher in fruit from low risk orchards at 2 weeks. As already mentioned, these compounds were not related to squalene, amyrin, or cycloartenol although there are a variety of phytosterol precursors with the same nominal mass.

In addition to the aforementioned squalene epoxidases, gene expression potentially related to phytosterol production included significantly higher levels of a transcript encoding HMG-CoA reductase (MDP000251253) in fruit from high risk orchards compared to those from low risk orchards beginning at 4 weeks of storage and continuing through the final measurement at 8 weeks. This enzyme catalyzes the first committed step of the MVA pathway and activity can be enhanced by feedback from products of enzymes in the sterol biosynthesis pathway [70, 71], although it would also precede MVA sesquiterpene synthesis. However, expression of sterol methyltransferase (SMT1; MDP000012279) was also higher during cold storage, although in high risk fruit, with the greatest increase between harvest and 2 weeks. There were no significant increases of sterol levels alongside expression of this gene, and overall, differential enhancement of free sterols appeared to be transient as none of the phytosterols/sterols or phytosterol/sterol conjugates were present in the group of combined significant compounds common to the analyses performed.

### Triacylglycerides and lipids levels are altered according to risk

At-harvest and throughout storage, levels of three partially identified triacylglycerides (TAG) were higher in fruit from all orchards that did not develop soft scald, while MGDG (C18:2, C18:2) was higher in fruit from high risk orchards. However, levels of this MGDG were also higher in fruit from low risk orchards following placement in cold storage (Fig. 9). MGDGs are a major lipid species comprising the thylakoid membrane and is synthesized in the inner plastid envelopes [72]. MGDG functions to establish and maintain photosystem II dimer structure [87]. Previous work revealed specific TAGs elevated in the brown necrotic tissue of soggy breakdown [16] but length, saturation and intermolecular organization of acyl components in that study may differ from present TAGs, which have not been fully characterized. It has been demonstrated that acyl moieties respond to temperature changes in apples, and differ with respect to physiological disorder outcome [15]. With respect to related gene expression, an oleosin transcript (MDP0000122458) was also elevated throughout storage fruit from high risk orchards. Oleosins function in the formation and stabilization of oil bodies containing TAGs [73] but are not typical in non-oil producing organs such as apple fruit peel. Nevertheless, the summation of these results indicate that TAGs and MGDG lipids may have a role in normal function of apple fruit cells following chilling, presumably in maintaining cellular or organellular membrane stability.

### Ethanol and ethyl esters are not impacted by soft scald risk

Ethanol and its acyl esters are produced by tissue affected by soggy breakdown, a disorder of the cortex that often occurs alongside with soft scald [16]. Levels of these compounds were not elevated in any treatment or at any time in the current study. Early in storage it is possible that levels of ethanol and ethyl esters had not accumulated to appreciable levels, but absence of significance later in storage may indicate they are not an important component of soft scald symptom development.

## Conclusions

Soft scald symptom development is the result of an adverse reaction to cold storage commonly employed to store apple fruit. Our results outline a process by which cold storage triggers a widespread shift in the transcriptome within the first 2 weeks followed by a shift in the metabolome. The processes and pathways involved in that shift were different depending on whether apples from a specific orchard developed soft scald. Factors that mitigated scald risk include not only fruit maturity, but also orchard cultural and environmental conditions not accounted for by this study. Processes and pathways indicating high risk were stress responses related to glutamate and phenolic metabolism as well as LOX and C6 volatile metabolism. Low risk was indicated by elevated levels of farnesol acyl esters and other isoprenoids as well as overexpression within the initial portions of isoprenoid biosynthesis and triterpenoid biosynthesis. In summary, environmental, cultural, and ontogenic factors all contributed to whether fruit remained healthy or developed soft scald and, accordingly, how metabolism was altered once fruit were placed in cold storage.

## Methods

### Sample collection

‘Honeycrisp’ apples (*Malus x domestica* Borkh.) were obtained from nine orchards distributed among the Lake Chelan/Brewster, Columbia Basin, and Yakima Valley growing regions in Washington State and the Hood River growing region of Oregon between September 2 and October 21, 2011. To determine the effects of harvest-timing on postharvest soft scald, fruit from one site was harvested at three different times, to represent an early, mid-, and late harvest (Orchard A). Fruit were stored in regular air at 1 °C. At harvest, and at 2, 4, and 8 weeks of storage, quality was assessed, and fruit peel tissue was sampled for metabolic and transcriptomic analysis. Soft scald and soggy breakdown incidence was visually assessed at each time point on 48 fruit.

### Fruit maturity assessment

Internal ethylene concentration (IEC), fruit firmness, titratable acidity, starch pattern index, weight, and color were assessed on 16 fruit at each time point. Peel background color was rated using a color wheel as a guide (United States Department of Agriculture, Standard Ground Color Chart for Apples and Pears in Western States). The same person rated all the fruit for the entire experiment.

Firmness was analyzed using a Mohr Digi-Test 1.25 penetrometer (Mohr & Associates, Richland, WA, USA) equipped with an 11-mm tip on one pared surface of each fruit. The maximum external fruit pressure (N), designated M1, indicates the firmness of the fruit from the peel boundary to a depth of 0.813 cm.

Starch hydrolysis was visually assessed on a full width tissue slice cut from the fruit equator using a 1–6 scale from the Washington Tree Fruit Research Commission (1 = no hydrolysis, all tissue black, 6 = hydrolysis complete, tissue white) [74] after staining with a 0.024 M I-KI solution. The same person visually assessed starch throughout the experiment.

Fresh juice, prepared using a Champion juicer (Plastaket Mfg., Lodi, CA, USA), was used to measure soluble solids content (SSC), using a handheld refractometer (ATAGO, Tokyo, Japan), and titratable acidity (TA) by titrating 10 mL juice with 0.1 M KOH to pH 8.2 using an autotitrator (TIM850, Radiometer Analytical, Copenhagen, Denmark).

IEC was measured by piercing the calyx end of the fruit with a wide-bore needle equipped with a rubber serum stopper. A 1 ml plastic syringe with a 1 inch needle (BD, Franklin Lakes, NJ, USA) was used to pierce the septum and slowly draw up 1 mL of gas sample from the core cavity. 0.5 mL of the gas was injected into a 5880A GC-FID (Hewlett-Packard, Avondale, PA, USA) equipped with a 50-cm, 0.32-cm-i.d. glass column packed with 80–100 mesh Porapak Q (Supelco, Bellafonte, PA, USA). The 5880 GC-FID was calibrated daily using 0.5 mL gas containing 9.01 μl (0.368 μmol) per L ethylene (Scotty Analyzed Gases, Bellefonte, PA, USA). The temperature of injector, oven and detector was 100 °C, 130 °C, and 200 °C, respectively. Gas flows for air, N_2_, and H_2_ were 300, 30, and 30 ml min^−1^, respectively.

For each sample replication, peel tissue was removed and flash frozen in liquid nitrogen. Samples were cryogenic ground in a N_2_ (*l*) cooled rotary mill (A 11 Basic Analytical Mill, IKA Works Inc., Wilmington, NC, USA) then returned to −80 °C for up to 4 months prior to metabolomic and transcriptomic analysis.

### Metabolic profiling

Five replications were assessed per orchard and time point. Metabolic extraction and instrumental analyses were carried out similar to Rudell et al. [13], Rudell et al. [15], and Leisso et al. [16]. Volatile metabolites present in ground tissue were assessed using a GC-MS volatile headspace sampling system. Sugars, sugar alcohols, organic acids, and amino acids were derivatized via a trimethylsilyl(oxime) protocol and assessed via GC-MS. Non-polar compounds, including phytosterols, *p*-coumaroyl esters, lipids, carotenoids, and chlorophylls, were extracted from tissue using an acetone/hexanes phase extraction protocol and analyzed utilizing accurate mass high performance liquid chromatograph equipped with a tandem quadrupole time-of-flight mass selective detector (HPLC- QTOF-MS). Compounds whose identity was confirmed by co-elution with authentic standards are indicated in Additional files 6: Table S2).

### RNA isolation and mRNA-seq library construction

For each sample replication, three fruit were peeled directly into liquid nitrogen. Transcriptomic profiling was performed on three composite (3 fruit each) biological replications for each orchard/time point combination using the protocol described by Gapper et al. [20]. Briefly, total RNA was extracted using a chloroform phase extraction and purified using a RNAeasy column (Qiagen). RNA-seq libraries were created using total RNA according to Zhong et al. [75]. mRNA was isolated from total RNA, fragmented and used as a template for cDNA synthesis via reverse transcription (Superscript III, Invitrogen). After the first strand of cDNA synthesis, the second strand was synthesized via a dNTP mix utilizing dUTP instead of dTTP. Ends of the double-stranded cDNAs were then repaired (Enzymatics), dA tailed by the Klenow enzyme (Enzymatics), and universal TruSeq adapters (Illumina) were ligated. After ligation, the second strand was digested by uracil DNA glycosylase (UDG) to produce strand-specific enrichment of the library. The UDG-digested cDNA was then used as a template to enrich the libraries by polymerase chain reaction (PCR) using primers containing the TruSeq barcodes, and the high-fidelity enzyme Phusion (NEB) with the conditions described in Gapper et al. [20]. 20 ng of the libraries were pooled for sequencing; 48 were multiplexed per sequencing reaction using an Illumina HiSeq 2000/2500 next-generation sequencer at the Weill Medicine School Sequencing Facility (Cornell University, New York City, NY, USA).

### Bioinformatics

Forty base pair single-end, strand-specific RNA-Seq reads were filtered by aligning rRNA and tRNA sequences to adapter using Bowtie, allowing two mismatches. High-quality reads were then aligned to an apple predicted cDNA list [76] using Tophat (allowing one seqment mismatch). After alignments, raw counts were normalized to reads per kilobase of exon model per million mapped reads (RPKM). Housekeeping gene expression was analyzed to confirm their constitutive expression (Additional file 8: Figure S5).

## Data analyses

The data analysis strategy is summarized in Table 2. Data analyses were performed to determine metabolites and gene expression that differ specifically at-harvest and 2 weeks following cold storage imposition among orchards with different soft scald incidence. Metabolites and transcripts differing at these time points according to eventual scald incidence may be indicative of scald risk. To achieve this, orchards were divided into “low-risk” and “high-risk” groups, based on soft scald incidence outcomes at 12 weeks (see Fig. 1 for disorder incidence; low risk: A_H1, C, M, N, P; high risk: A_H2, G, F, K, A_H3, L [fruit from orchard A_H2 developed soft scald at 12 weeks, and therefore was classified as high risk]). Any incidence of soft scald resulted in the categorization of the orchard as a “high-risk” orchard. Even orchards with low incidence of soft scald were considered “high-risk” as any incidence can result in economic loss.

**Table 2:** Summary of data analyses procedure for both transcriptomic and metabolomic data of ‘Honeycrisp’ apple fruit to predict postharvest soft scald risk

Data type	Timepoints	Objective	Analysis
Transcriptomic, normalized	0–8 wk	Latent trends	Principal components analysis (PCA)
normalized	0–8 wk	Differential expression in the multivariate case	ANOVA-simultaneous component analysis
raw counts	0 wk	Pre-harvest environmental effects	RobiNA edge R differential expression according to soft scald risk^a^
raw counts	2 wk	Effects of chilling	RobiNA edge R differential expression according to soft scald risk^a^
	-	Functional characterization of significant transcripts	Gene Ontology overrepresentation analysis
Metabolomic, means centered, standard deviation squared	0–8 wk	Latent trends	Principal components analysis (PCA)
	0–8 wk	Differential expression in the multivariate case	ANOVA-simultaneous component analysis
	0 wk	Pre-harvest environmental effects	*t*-test/ Pearson’s correlation + Ward clustering algorithm heatmap visualization
	2 wk	Effects of chilling	*t*-test/ Pearson’s correlation + Ward clustering algorithm heatmap visualization
Transcriptomic and metabolomic, normalized and set to a common scale by means centering	0–8 wk	Correlation among expressed genes and metabolites	Pearson’s correlation via Cytoscape Expression Correlation plug-in at *r* = │0.95│, followed by network subtraction with the Network Analyzer plug-in

Data sets were analyzed separately to find differences between high and low risk orchards at-harvest, after 2 weeks cold storage, and overall

^a^Soft scald risk assigned categorically as “low risk”/“high risk” based on disorder incidence at 12 weeks of storage. “Low risk”, no fruit exhibited soft scald symptoms. “High risk”, the presence of any fruit with soft scald symptoms

The other intended outcome of data analyses was to determine metabolite and gene expression trends over the course of cold storage, specifically assessing whether particular biochemical pathways were differentially up- or down-regulated according to orchard susceptibility. For this purpose, ANOVA simultaneous component (ASCA) was applied [77–79]. This analysis combines elements of multivariate statistics to delineate the primary latent variables, or trends in the data, along with determining which metabolites or RNA sequences differ according to discriminant classification, which was orchard risk for soft scald.

### Comparative analysis of gene expression

To define how known experimental factors were involved in differential gene expression among orchards that differ in soft scald susceptibility, principal components analysis (PCA) of means centered/standard deviation squared normalized RNA-seq data was performed using Unscrambler (Camo Inc., Woodbridge, NJ, USA), and ANOVA simultaneous component analysis (ASCA) was performed using MetaboAnalyst [77], with orchards categorized according to susceptibility (0,1; low and high risk) and time (0, 2, 4, 8 weeks). For ASCA, data were first filtered to remove gene models whose average value was less than 4 RPKM (reducing to ~23,400 gene models), and then further filtered to 5000 gene models using non-parametric relative standard deviation to retain transcripts with the greatest variability among the conditions. Expression data were mean-centered, divided by their standard deviation and, then, analyzed as time series repeated measures. The model was validated using permutation test statistics, and was significant at *p* <0.005 for risk, time, and risk*time (leverage = 0.90). Transcripts which were significant in the model were determined for risk, time and risk*time.

### Comparison of at-harvest gene expression

To determine the differences among orchards at harvest, expression data were analyzed using RobiNA [80]. Data from nine orchards were used, one with three harvests. Data were imported as a raw counts table, and differential expression was analyzed with the edgeR package [81], using a *p*-value cutoff of *p* <0.05, corrected for false discovery rate (FDR) [82]. Input was filtered to remove transcripts with a less than two-fold change. Dispersion was estimated globally.

### Comparison of chilling-related gene expression

To reveal the impact of chilling, 2 week RNA-seq data were also analyzed using RobiNA [80], applying the edgeR package [81] using a cutoff of *p* <0.05, corrected by false discovery rate (FDR) [82]. Data were imported as a raw counts table. A subset of samples from orchards were available for 2 week analysis (orchards A [3 harvests], G, M).

### Functional analysis of significant gene expression

Gene models significant for consistent differential change over the storage period in ASCA as well as at-harvest and 2 week DEGs were analyzed using gene ontology (GO) [83, 84] to perform an overrepresentation analysis according to comparatively elevated levels in fruit from orchards with low risk for soft scald or fruit from orchards susceptible to soft scald. Significant genes were uploaded to Cytoscape 2.8.3 [85, 86] and analyzed via the BinGO 2.44 plug-in [83]. The ontology file used was GO-basic.obo, download from Geneontology.org (03/03/2015). The GO annotation was obtained for *Malus* x *domestica* from the genome database for Rosaceae [23, 76]. For the analysis, the whole annotation was used as a reference set. Up-regulated genes were assessed for overrepresentation using the BinGO plugin v 2.44 [83] for Cytoscape v 2.8.3 [85, 86]. Overrepresentation was assessed using a hypergeometric test at *p* <0.05, corrected by false discovery rate (FDR) for each of the GO categories “biological process”, “cellular component”, and “molecular function” [84]. Overrepresented categories were visualized as nodes.

### Comparative analysis of metabolites

To summarize metabolism contributing to differences among orchards, both principal components analysis PCA and ASCA were performed on 833 metabolites. Orchards were categorized by risk (0, 1; low and high risk) and time (0, 2, 4, 8 weeks) using 9 orchards [one orchard (A) with three harvest dates]. Data were first scaled using mean-centering and dividing by the standard deviation. Data were analyzed as time series repeated measures. The model was validated using permutation test statistics at a significance of *p* <0.005 for risk, time, and risk*time. Identified metabolites that were significant in the model were determined (leverage = 0.90 and alpha = 0.001).

### At harvest metabolic profiling

To determine differences among orchards according to soft scald risk at the time of harvest, at-harvest metabolite data were analyzed using a *t*-test (*p* < 0.05) and cluster analysis (Pearson’s *r*, Ward’s clustering algorithm) and visualized using a heatmap generated using MetaboAnalyst [77, 78]. Prior to analysis, data were normalized using autoscaling procedures (mean-centered and divided by the standard deviation of each variable). Data from nine orchards were used, one with three harvests.

### Chilling response at 2 weeks in the metabolome

To determine the differential effects of chilling, 2 week metabolite data were analyzed using a *t*-test (*p* < 0.05) and visualized using a cluster analysis (Pearson’s *r*, Ward’s clustering algorithm) to organize a heatmap using online software from MetaboAnalyst [85, 86]. Although more data were available, in order to directly correspond with the RNA-seq data, the 2 week data from three orchards were used to compare with the dataset used for gene expression analysis, one orchard had three harvests and differing soft scald outcomes were based on expected differences of maturity.

### Correlation and network analysis of gene expression and metabolite data

Metabolites and genes with increased levels/expression either in fruit peel from low risk or high risk orchards were normalized using means centering. Low risk and high risk correlation networks were created using Pearson’s correlation coefficient (*r* ≥ |0.95|) in Cytoscape [85, 86]. Network subtractions (high risk – low risk; low risk – high risk) were performed using Network Analyzer, a pre-installed Cytoscape app [85, 86], to determine highly correlated element that differentiated fruit peel from high risk or low risk orchard given all experimental factors.

## Additional files

Additional file 1: Table S1. Quality and maturity of fruit from ‘Honeycrisp’ orchards used for the metabolomic (all orchards) and transcriptomic (orchards A_H1, A_H2, A_H3, M, and P) analyses. (DOCX 34 kb)
Additional file 2: Figure S1. At-harvest expression (RPKM) heatmap genes associated with apple ripening. (DOCX 228 kb)
Additional file 3: Figure S2. Up-regulated differentially expressed genes common among 0 W (at harvest), 2 W (2 weeks), and ASCA results for fruit from low risk and high risk orchards. (DOCX 272 kb)
Additional file 4: Figure S3. Subtractive (high risk – low risk) correlation network of highly correlated expressed genes and metabolites. (DOCX 373 kb)
Additional file 5: Figure S4. Subtractive (high risk – low risk) correlation network of highly correlated expressed genes and metabolites. Expressed genes are indicated by green circles while metabolites are indicated by blue squares. A subnetwork containing both highly correlated gene expression and metabolite levels. (DOCX 288 kb)
Additional file 6: Table S2. (A,B,C). Identified metabolites detected apple fruit peel. (DOCX 38 kb)
Additional file 7: Protocol S1. Farnesyl oleate, farnesyl linolenate, and farnesyl linoleate standard synthesis. (DOCX 14 kb)
Additional file 8: Figure S5. Expression levels (RPKM) of *Malus domestica* housekeeping genes from Bowen et al. (2015) in RNA-seq analyses. (DOCX 174 kb)
