# Supplementary material for: Gene expression and metabolism preceding soft scald, a chilling injury of ‘Honeycrisp’ apple fruit
Source: BMC Genomics. 2016 Oct 12;17:798. doi: 10.1186/s12864-016-3019-1 (PMC5062943; doi:10.1186/s12864-016-3019-1)
Supplement: Additional file 1: Table S1. — Quality and maturity of fruit from ‘Honeycrisp’ orchards used for the metabolomic (all orchards) and transcriptomic (orchards A_H1, A_H2, A_H3, M, and P) analyses. (DOCX 34 kb) [file 12864_2016_3019_MOESM1_ESM.docx]

Table S1. Quality and maturity of fruit from ‘Honeycrisp’ orchards used for the metabolomic (all orchards) and transcriptomic (orchards A_H1, A_H2, A_H3, M, and P) analyses. For orchard A, there were 3 successive harvests, indicated by _H1, _H2, and _H3. Values followed by the same letter are not different [type III sums of squares in proc GLM (SAS Institute, Cary, NC, USA) and mean separation using Tukey's HSD *post hoc* test]. Shading indicates orchards that developed soft scald during storage (see also Figure 1).

| **Orchard** | **WSU^1^ AgWeatherNet weather station^2^** | **Harvest date** | **Growing degree days (GDD)^3^** | **Chilling hours^4^** | **Weight (g)** | **Firmness (Max. Ext.; N)** | **Soluble solids (˚Bx)** | **Background color (1-4)** | **Starch index**  **(1-6)^5^** | **Titratable acidity**  **(% malic acid)** | **IEC (µl/L)^6^** | **Streif index^7^** |
| --- | --- | --- | --- | --- | --- | --- | --- | --- | --- | --- | --- | --- |
| A _H1 | Gleed | 9/2/2011 | 1444 | 11 | 236.5 cd | 71.6 a | 12.2 d | -- | 1.2 f | 0.56 a | 0.33 b | 3.67 a |
| C | Mattawa | 9/15/2011 | 2368 | 3 | 268.4 bcd | 56.5 de | 13.3 b | 3.6 ab | 5.3 bdc | 0.43 d | 16.62 a | 0.60 b |
| A _H2 | Gleed | 9/19/2011 | 1708 | 36 | 320.5 a | 59.2 cde | 13.7 a | 3.2 b | 5.1 d | 0.54 a | 14.65 a | 0.64 b |
| G | Cowiche | 9/26/2011 | 2051 | 62 | 236.0 cd | 63.2 bc | 13.8 a | 2.7 c | 4.6 e | 0.56 a | 3.67 b | 0.75 b |
| F | FishHook | 9/26/2011 | 2283 | 46 | 300.8 ab | 60.1 bcd | 12.8 c | 3.6 ab | 5.1 cd | 0.51 b | 5.46 b | 0.69 b |
| K | Frenchmen Hills | 10/3/2011 | 2036 | 99 | 280.4 abc | 55.2 e | 13.0 c | 3.5 ab | 5.6 abc | 0.35 f | 2.11 b | 0.57 b |
| A _H3 | Gleed | 10/5/2011 | 1829 | 138 | 300.5 ab | 58.7 cde | 13.8 a | 3.9 a | 5.8 a | 0.46 c | 2.34 b | 0.55 b |
| L | Chelan South | 10/6/2011 | 2406 | 35 | 256.4 bcd | 60.9 bcd | 12.2 d | 3.8 a | 5.2 bcd | 0.43 d | 3.54 b | 0.72 b |
| M | Cowiche | 10/7/2011 | 2104 | 147 | 266.7 bcd | 61.8 bc | 13.5 ab | 3.1 b | 5.2 dc | 0.47 c | 4.19 b | 0.66 b |
| N | Underwood | 10/18/2011 | 1686 | 149 | 191.4 e | 61.4 bcd | 11.7 e | 2.2 d | 5.7 ab | 0.41 de | 3.84 b | 0.69 b |
| P | Boyd District | 10/21/2011 | 1917 | 367 | 222.9 de | 64.5 b | 11.6 e | 3.6 ab | 6.0 a | 0.39 e | 0.92 b | 0.70 b |
| Significance^8^ |  |  |  |  | * | * | * | * | * | * | * | * |

^1.^ WSU, Washington State University

^2.^ Weather.wsu.edu

^3.^ Base 10 ˚C from 1 August 2011

^4.^ Base 10 ˚C from 1 August 2011

^5.^ Washington Tree Fruit Research Commission starch scale for 'Honeycrisp'

^6.^ IEC, internal ethylene concentration

^7.^ firmness/SSC*starch index

^8.^ *, *p* <0.0001
