# Supplementary material for: Gene expression and metabolism preceding soft scald, a chilling injury of ‘Honeycrisp’ apple fruit
Source: BMC Genomics. 2016 Oct 12;17:798. doi: 10.1186/s12864-016-3019-1 (PMC5062943; doi:10.1186/s12864-016-3019-1)
Supplement: Additional file 2: Figure S1. — At-harvest expression (RPKM) heatmap genes associated with apple ripening. (DOCX 228 kb) [file 12864_2016_3019_MOESM2_ESM.docx]

Figure S1. At-harvest expression (RPKM) heatmap genes associated with apple ripening (A). SS = soft scald, HR = high risk, LR = low risk. Full accession numbers are listed in (B). Two-factor ANOVA (*p* <0.05, with false discovery rate multiple testing correction) indicates no significance among orchards or harvests (C).

A

| **Gene** | **MDP** |
| --- | --- |
| ACO1 | MDP0000195885 |
| B-GAL | MDP0000416548 |
| PG1 | MDP0000326734 |
| B-XyL | MDP0000140483 |
| AF | MDP0000199152 |
| AAT1 | MDP0000637737 |
| B-amylase | MDP0000196961 |
| ACS1 | MDP0000370791 |
| ACS3A | MDP0000145123 |
| ACS3B2 | MDP0000286209 |
| ACS3B3 | MDP0000248461 |
| ACS3C | MDP0000373968 |
| ACS5A | MDP0000435100 |
| ACS5B | MDP0000923426 |


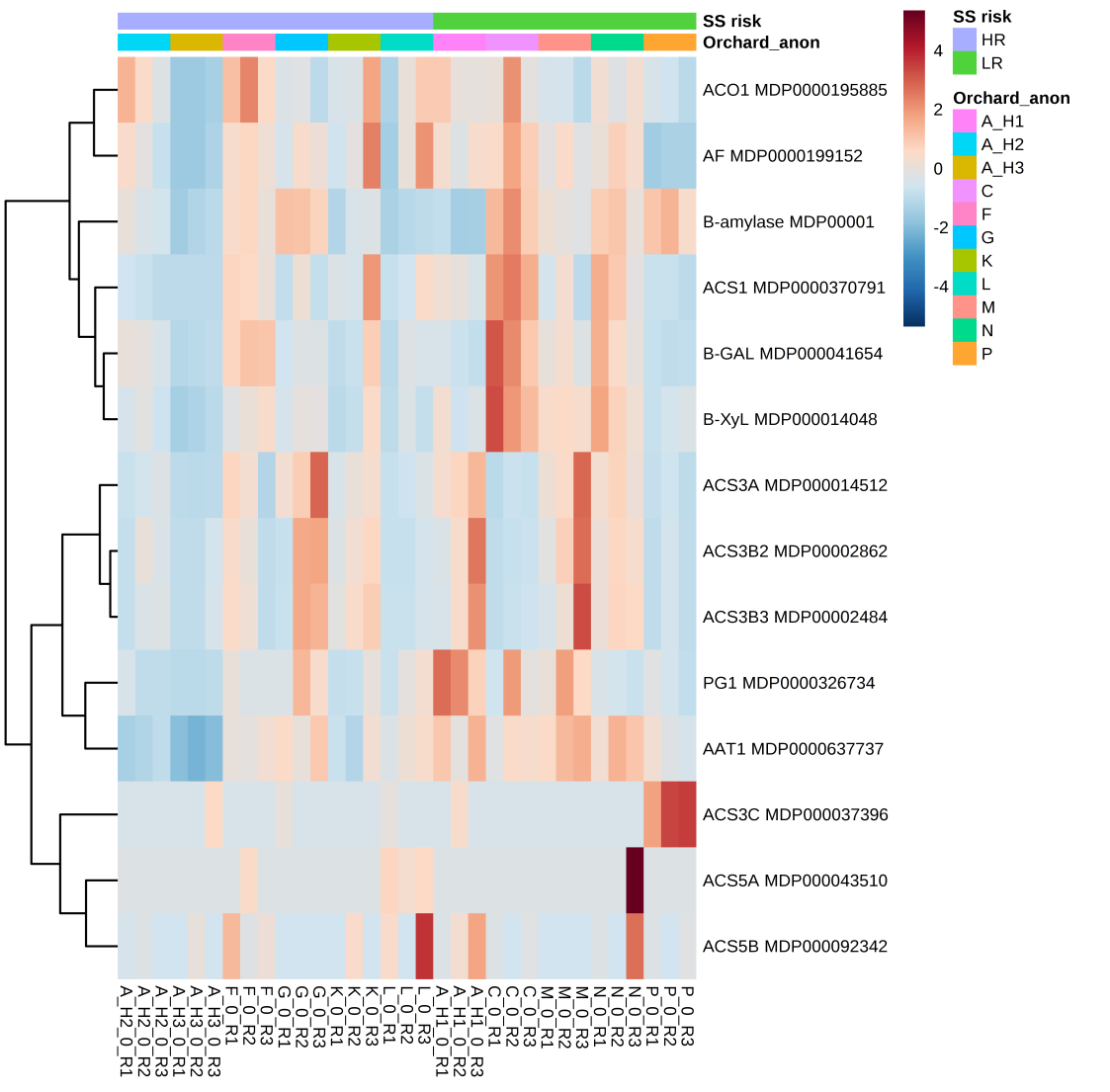


B


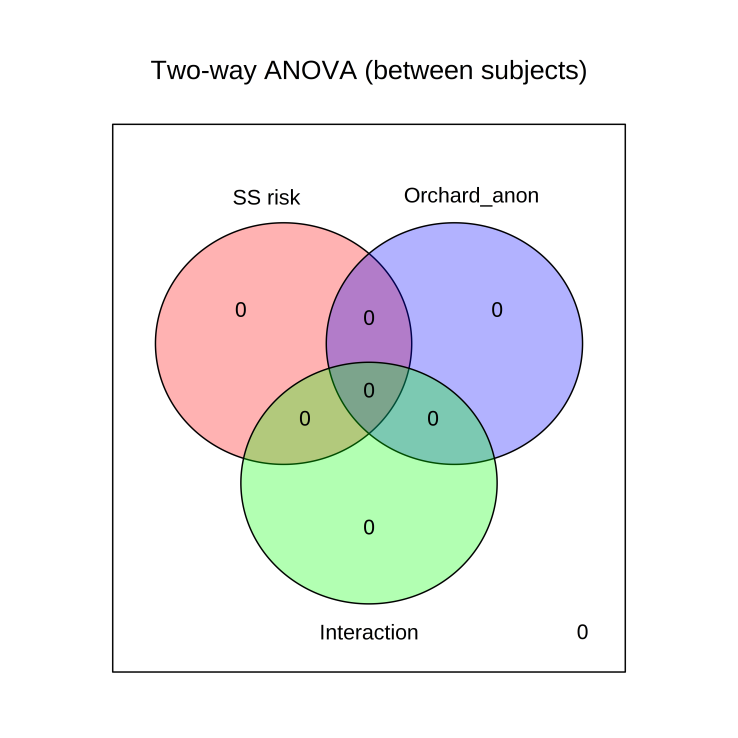


C
