# Supplementary material for: Gene expression and metabolism preceding soft scald, a chilling injury of ‘Honeycrisp’ apple fruit
Source: BMC Genomics. 2016 Oct 12;17:798. doi: 10.1186/s12864-016-3019-1 (PMC5062943; doi:10.1186/s12864-016-3019-1)
Supplement: Additional file 3: Figure S2. — Up-regulated differentially expressed genes common among 0 W (at harvest), 2 W (2 weeks), and ASCA results for fruit from low risk and high risk orchards. (DOCX 272 kb) [file 12864_2016_3019_MOESM3_ESM.docx]

Figure S2. Up-regulated differentially expressed genes common among 0W (at harvest), 2W (2 weeks), and ASCA results for fruit from A) low risk and B) high risk orchards. Over representation analysis of DEGs at harvest and 2 weeks according to risk [C) low risk, D) high risk] was performed using the BinGO app (Cytoscape). Colored nodes represent GO terms significantly overrepresented; the intensity of orange color indicates *p*-value and size represents the number of genes. Inset tables contain the genes corresponding to significant GO terms.

Supplemental figure 1 (continued). Inset tables are RNA gene models and annotation corresponding to significant GO terms in low risk (E) and high risk (F) orchards.

E. Low risk

| Gene model | Annotation |
| --- | --- |
| MDP0000824044 | Phytosulfokine |
| MDP0000145144 | Phytosulfokine |

F. High risk

| Gene model | Annotation |
| --- | --- |
| MDP0000121476 | Myb transcription factor |
| MDP0000132919 | zinc finger (C2H2 type) family protein |
| MDP0000145050 | Myb-related protein Myb4 |
| MDP0000149492 | RING-H2 finger protein |
| MDP0000225132 | Zinc finger, RING/FYVE/PHD-type |
| MDP0000237761 | Zinc finger, RING/FYVE/PHD-type |
| MDP0000273866 | UDP-glucuronosyl/UDP-glucosyltransferase |
| MDP0000274301 | Myb transcription factor/ Myb domain, DNA-binding |
| MDP0000276057 | UDP-glucuronosyl/UDP-glucosyltransferase |
| MDP0000278681 | Myb transcription factor/ Myb domain, DNA-binding |
| MDP0000280105 | Cytochrome P450, E-class group I |
| MDP0000288465 | AP2/ERF domain |
| MDP0000294168 | Fatty acid hydroxylase |
| MDP0000304625 | UDP-glucuronosyl/UDP-glucosyltransferase |
| MDP0000308543 | UDP-glucuronosyl/UDP-glucosyltransferase |
| MDP0000361351 | Aldo/keto reductase subgroup/NADP-dependent oxidoreductase domain |
| MDP0000408705 | Aldo/keto reductase subgroup/NADP-dependent oxidoreductase domain |
| MDP0000411091 | zinc finger (C2H2 type) family protein |
| MDP0000639894 | NADP-dependent D-sorbitol-6-phosphatedehydrogenase |
| MDP0000715270 | Oxoglutarate/ion-dependent oxygenase (gibberellin 20 oxidase 1) |
| MDP0000737001 | Oxoglutarate/ion-dependent oxygenase (Naringenin,2-oxoglutarate 3-dioxygenase) |
| MDP0000742771 | Myb transcription factor |
| MDP0000818877 | NADP-dependent D-sorbitol-6-phosphate dehydrogenase |
| MDP0000911051 | Myb transcription factor |
