# Supplementary material for: Gene expression and metabolism preceding soft scald, a chilling injury of ‘Honeycrisp’ apple fruit
Source: BMC Genomics. 2016 Oct 12;17:798. doi: 10.1186/s12864-016-3019-1 (PMC5062943; doi:10.1186/s12864-016-3019-1)
Supplement: Additional file 5: Figure S4. — Subtractive (high risk – low risk) correlation network of highly correlated expressed genes and metabolites. Expressed genes are indicated by green circles while metabolites are indicated by blue squares. A subnetwork containing both highly correlated gene expression and metabolite levels. (DOCX 288 kb) [file 12864_2016_3019_MOESM5_ESM.docx]

Figure S4. Subtractive (high risk – low risk) correlation network of highly correlated expressed genes and metabolites (A). Expressed genes are indicated by green circles while metabolites are indicated by blue squares. A subnetwork containing both highly correlated gene expression and metabolite levels (B).
