# Supplementary material for: Gene expression and metabolism preceding soft scald, a chilling injury of ‘Honeycrisp’ apple fruit
Source: BMC Genomics. 2016 Oct 12;17:798. doi: 10.1186/s12864-016-3019-1 (PMC5062943; doi:10.1186/s12864-016-3019-1)
Supplement: Additional file 6: Table S2. — (A,B,C). Identified metabolites detected apple fruit peel. (DOCX 38 kb) [file 12864_2016_3019_MOESM6_ESM.docx]

**Table S2 (A,B,C)**. Identified metabolites detected apple fruit peel using (A) volatile headspace, (B) methanolic extraction and TMS derivatization (polar), and (C) acetone extraction (non-polar), followed by assessment of relative levels on GC-MS (for A and B) and LC-MS ( for C). Metabolites were identified by co-elution with authentic standards are listed according to extraction procedure and mass spectral tag (retention index or time, and target ion).

| **Table S1 (A). Volatile headspace extraction; analysis of extracts on GC-MS** | | | | | |  | | |  |
| --- | --- | --- | --- | --- | --- | --- | --- | --- | --- |
| **Metabolite** | **Abbreviation** | **Mass spectral tag**  **(retention index, target ion)** | **Co-elution standard source** | **Metabolite** | **Abbreviation** | | **Mass spectral tag**  **(retention index, target ion)** | **Co-elution standard source** | |
| (E) 2-heptenal | 2HeptHO | 961.5, 83 | Sigma Aldrich*^a^* | ethanol | EtOH | | 526.3, 45 |  | |
| (E) 2-octenal | 2OctHO | 1071.0, 83 | Sigma-Aldrich | ethyl 2-methylbutanoate | Et2MeB | | 851.3, 57 | Sigma-Aldrich | |
| (E)-2-hexenal | 2HHO | 855.6, 98 | Sigma-Aldrich | ethyl acetate | EtA | | 617.5,88 | Sigma-Aldrich | |
| (E,E) 2,4-hexadienal | 2,4Hex | 914.8, 81 | Sigma-Aldrich | ethyl butanoate | EtB | | 805.1, 43 | Sigma-Aldrich | |
| (E,E) α-farnesene | EEaFarn | 1509.5, 119 | purified/extracted*^b^* | ethyl hexanoate | EtH | | 999.3, 88 | Sigma-Aldrich | |
| (Z) 2-hexen-1-ol | 2HOH | 869.4, 57 | Sigma-Aldrich | ethyl pentanoate | EtPent | | 902.3, 85 | Sigma-Aldrich | |
| (Z) 3-hexen-1-ol | 3HOH | 854.4, 82 | Sigma-Aldrich | ethyl propanoate | EPro | | 718.5, 57 | Sigma-Aldrich | |
| (Z,E) α-farnesene | ZEaFarn | 1496.6, 93 | purified/extracted*^b^* | heptanal | HeptHO | | 903.3, 43 | Sigma-Aldrich | |
| 1-butanol | BOH | 672.8, 56 | Sigma-Aldrich | hexanal | HHO | | 804.9. 41 | Sigma-Aldrich | |
| 1-hexanol | HOH | 871.8, 56 | Sigma-Aldrich | hexyl 2-methylbutenoate | H2MButen | | 1331.2, 109 | Sigma-Aldrich | |
| 1-pentanol | PentOH | 772.4, 42 | Sigma-Aldrich | hexyl 2-methyl butyrate | H2MeB | | 1235.8, 103 | Sigma-Aldrich | |
| 1-propanol | POH | 583.3, 31 | Sigma-Aldrich | hexyl acetate | HA | | 1013.2, 43 | Sigma-Aldrich | |
| 2-butanol | 2BOH | 616.2, 45 | Sigma-Aldrich | hexyl butanoate | HB | | 1190.4, 99 | Sigma-Aldrich | |
| 2-methyl-1-butanol acetate | 2MeBA | 880.3, 70 | Sigma-Aldrich | hexyl hexanoate | HH | | 1383.8, 117 | Sigma-Aldrich | |
| 2-methyl-1-propanol | 2MPHOH | 629.3, 43 | Baker*^c^* | hexyl pentanoate | HPen | | 1287.3, 85 | synthesized*^d^* | |
| 2-methylbutanol | 2MeBOH | 741.9, 57 | Sigma-Aldrich | hexyl propanoate | HPro | | 1104.2, 57 | Sigma-Aldrich | |
| 2-methylbutylhexanoate | 2MeBH | 1254.8, 99 | synthesized*^d^* | methoxybenzene | MxBenz | | 902.2, 108 | Sigma-Aldrich | |
| 2-methylpropylbutanoate | 2MePB | 956.4, 71 | synthesized*^d^* | methyl 2-methylbutyrate | Me2MeB | | 781.7, 88 | Sigma-Aldrich | |
| (E) 2-pentenal | 2PentHO | 759.5, 83 | Alfa Aesar*^e^* | methyl 2-methylpropanoate | Me2MeP | | 687.0, 71 | synthesized*^d^* | |
| 2-propanol | 2ProOH | 531.6, 45 | Fisher*^f^* | methyl acetate | MeA | | 532.6, 74 | Sigma-Aldrich | |
| 6-methyl-5-hepten-2-ol | 6MHol | 994.5, 95 | Sigma-Aldrich | methyl alcohol | MeOH | | 491.6, 31 | Fisher | |
| 6-methyl-5-hepten-2-one | 6MHO | 988.4, 55 | Sigma-Aldrich | methyl butanoate | MeB | | 729.2, 74 | Sigma-Aldrich | |
| acetaldehyde | AHO | 471.0, 44 | Fisher | methyl hexanoate | MeH | | 925.4, 74 | Sigma-Aldrich | |
| acetic acid | AOOH | 706.2, 60 | Mallinckrodt *^g^* | methyl propionate | MePro | | 632.8, 57 | Sigma-Aldrich | |
| acetone | AcO | 509.5, 58 | Sigma-Aldrich | nonanal | NonHO | | 1116, 57 | Sigma-Aldrich | |
| benzaldehyde | BenzHO | 965.6, 106 | Sigma-Aldrich | pentanal | PentHO | | 701.9, 44 | Sigma-Aldrich | |
| β-farnesene | Bfarn | 1457.1, 69 | TCI*^h^* | pentyl butyrate | PentB | | 1093.4, 71 | Sigma-Aldrich | |
| butyl 2-methylbutyrate | B2MeB | 1041.9, 57 | Sigma-Aldrich | pentyl hexanoate | PentHO | | 1286.3, 117 | Sigma-Aldrich | |
| butyl acetate | BA | 817.6, 43.1 | Sigma-Aldrich | pentyl acetate | PentA | | 914.9, 70 | Sigma-Aldrich | |
| butyl butyrate | BB | 995.7, 71 | Sigma-Aldrich | propyl butanoate | PB | | 899.4, 71 | Sigma-Aldrich | |
| butyl hexanoate | BHO | 1190.0, 99 | synthesized*^d^* | propyl hexanoate | PH | | 1094.0, 99 | Sigma-Aldrich | |
| butyl propanoate | BP | 909.4, 57 | synthesized*^d^* | propyl propionate | PP | | 813.0, 57 | Sigma-Aldrich | |
| 4-allyl anisole | Estra | 1202.4, 148 | Sigma-Aldrich |  |  | |  |  | |

a. Sigma-Aldrich Co. LLC, St. Louis, MO.

b. Purification and identification were performed as described in Rudell et al. 2005.

c. J.T. Baker, Hayward, CA.

d. Synthesis and purification were performed similar to Fischer et al. 1895

e. Alfa Aesar, Ward Hill, MA.

f. Fisher Scientific, Waltham, MA.

g. Mallinckrodt Baker, Phillipsburg, NJ.

h. TCI, Portland, OR.

| **Table S2 (B) Methanol extraction and TMS derivatization; analysis of extracts on GC-MS** | | | |  |  |  |  |  |
| --- | --- | --- | --- | --- | --- | --- | --- | --- |
| **Metabolite** | **Abbreviation** | **Mass spectral tag**  **(retention index, target ion)** | **Co-elution standard source** | | **Metabolite** | **Abbreviation** | **Mass spectral tag**  **(retention index, target ion)** | **Co-elution standard source** |
| (-) epicatechin | Epicat | 2886.2, 368 | Sigma-Aldrich | | hydroxy-proline | proOH | 1530.6, 230 | Sigma-Aldrich |
| (+/-) catechin | Cat | 2905.9, 368 | Sigma-Aldrich*^b^* | | myo-inositol | Ino | 1832.7, 273 | Sigma-Aldrich |
| 1-aminocyclopropane-1-carboxylic acid | ACC | 1216.9, 202 | Sigma-Aldrich | | isoleucine | Ile | 1296.7, 158 | Sigma-Aldrich |
| 5-oxo-proline | Oxopro | 1529.7, 156 | Sigma-Aldrich | | leucine | Leu | 1276.5, 158 | Sigma-Aldrich |
| γ-aminobutryic acid | GABA | 1535.5,304 | Sigma-Aldrich | | linoleic acid | Lin | 2210.7, 337 | C18:2OOH |
| adonitol | ribOH | 1745.2, 217 | Sigma-Aldrich | | maleic acid | Maleic | 1307.9, 245 | Sigma-Aldrich |
| L-alanine | Ala | 112.0, 116 | Sigma-Aldrich | | malic acid | Malic | 1495.0, 335 | Sigma-Aldrich |
| asparagine | Asn | 1601.2, 116 | Sigma-Aldrich | | malonic acid | Malon | 1208.6, 233 | Fisher |
| L-aspartic acid | Asp | 1524.8, 232 | Sigma-Aldrich | | mucic acid | Mucic | 2079.2, 333 | Fluka |
| β-alanine | BAla | 1432.2, 248 | Sigma-Aldrich | | norvaline | Norval | 1241.0, 144 |  |
| chlorogenic acid | 5caffquin | 3146.7, 345 |  | | phenylalanine | Phe | 1555.8, 218 | Sigma-Aldrich |
| citramalic acid | Citmal | 1482.4, 247 | Sigma-Aldrich | | phosphoric acid | PO4 | 1279.9, 299 | J.T. Baker |
| citric acid | Cit | 1840, 273 | Sigma-Aldrich | | proline | Pro | 1529.7, 156 | Sigma-Aldrich |
| dodecanoic acid | C12OOH | 1648.8, 257 | Sigma-Aldrich | | pyruvic acid | Pyr | 1060.9, 174 | Sigma-Aldrich |
| eicosanoic acid | EicA | 2434.5, 369 | Sigma-Aldrich | | quinic acid | Quin | 1889.5, 345 | Sigma-Aldrich |
| erythritol | EryOH | 1517.3, 217 |  | | raffinose | Raff | 3492.6, 361 | Sigma-Aldrich |
| erythrose | Eryth2 | 1463.3, 205 | Fluka*^c^* | | rhamnose | Rhamn | 1737, 117 | Sigma-Aldrich |
| fructose | Fruct | 1911.1, 307*^a^* | Baker*^d^* | | ribose | Rib | 1696.2, 307 |  |
| fructose 6-phosphate | F6P | 2352.1, 315 | Fluka | | serine | Ser | 1364.8, 204 | Sigma-Aldrich |
| fumaric acid | Fum | 1349.0, 245 | Sigma-Aldrich | | sorbitol | Sorb | 1369.1, 319 | Sigma-Aldrich |
| gluconic acid | gluconic | 2040.8, 292 | Sigma-Aldrich | | succinic acid | Succ | 1316.1, 319 | Sigma-Aldrich |
| glucose | Gluc | 1930.8,205/217*^a^* | Baker | | sucrose | Suc | 2702.9, 361 | Sigma-Aldrich |
| glucose-6-phosphate | G6P | 2366, 387 | Sigma-Aldrich | | threonic acid | Threonic | 1393.7, 218 | Fluka |
| glutamic acid | Glu | 1624.8, 246 | Pierce | | threonine | Thr | 1393.7, 218 | Sigma-Aldrich |
| glyceric acid | Glyceric | 1336, 147 | Sigma-Aldrich | | L-valine | Val | 1221.7, 144 | Sigma-Aldrich |
| glycerol | Glycerol | 1279.8, 218 | Sigma-Aldrich | | xylitol | xylOH | 1741.8, 307 | Sigma-Aldrich |
| glycerol 3-phosphate | G3P | 1775.9, 299 | Sigma-Aldrich | | xylose | Xyl | 1674.0, 217 | Mann Research*^f^* |
| glycine | Gly | 1308.7,174 | Fisher*^e^* | |  |  |  |  |

a. Results from two peaks pooled.

b. Sigma-Aldrich Co. LLC, St. Louis, MO.

c. Fluka Chemical Co., Milwaukee, WI.

d. J.T. Baker, Hayward, CA.

e. Fisher Scientific, Waltham, MA.

f. Mann Research Center, Port St. Lucie, FL.

| **Table S2 (C) Acetone/HEPES extraction; analysis of extracts on LC-MS** | | |  |
| --- | --- | --- | --- |
| **Metabolite** | **Abbreviation** | **Mass spectral tag**  **(retention time, target ion)** | **Co-elution standard source** |
| 1,2-dilinoleoyl-3-oleoyl-rac-glycerol | LLO | 26.3, 881.7574 *^f^* | Sigma-Aldrich*^e^* |
| 1,2-dilinoleoyl-3-palmitoyl-rac-glycerol | LLP | 26.1, 855.7454 *^f^* | Sigma-Aldrich |
| 2, 6, 10-trimethyldodeca-2,7(E),9(E),11-tetra-6-ol | CTol*^a^* | 4.6, 203.1789 | Purified/identified^a^ |
| β-carotene | Caro | 22.8, 537.4456 | Fluka*^b^* |
| β-sitosteryl (6’-O-linolenoyl) β-D-glucoside | sitoLnG | 21.5, 397.3845 | synthesized*^c^* |
| β-sitosterol | sito | 18.7, 397.3830 | ChromaDex*^d^* |
| β-sitosteryl (6’-O-linoleoyl) β-D-glucoside | sitoLG | 22.0, 397.3841 | synthesized*^c^* |
| β-sitosteryl (6’-O-stearate) β-D-glucoside | sitoSG | 23.5, 397.3843 | synthesized*^c^* |
| β-sitosteryl linolenate | sitoLn | 26.7, 397.3825 | synthesized*^c^* |
| β-sitosteryl linoleate | sitoL | 28.1, 397.3855 | synthesized*^c^* |
| β-sitosteryl palmitate | sitoP | 30.0, 397.3827 | synthesized*^c^* |
| campesteryl (6’-O-linolenoyl) β-D-glucoside | CampG | 21.9, 383.3662 | synthesized*^c^* |
| chlorophyll a | ChlA | 19.3, 893.5432 | Sigma-Aldrich |
| chlorophyll b | ChLB | 17.9, 907.5218 | Sigma-Aldrich |
| farnesyl linoleate | sestc546 | 20.3, 205.1952 | synthesized |
| farnesyl linolenate | sestc644 | 19.5, 205.1943 | synthesized |
| farnesyl oleate | sestc389 | 21.3, 205.1947 | synthesized |
| glycerol trilinoleate | LLL | 25.5,879.7453 *^f^* | Sigma-Aldrich |
| lutein | Lut | 13.7, 551.4288 | Sigma-Aldrich |
| *p*-coumaryl stearate | pCstear | 16.5, 133.0643 | BDW Standard^g^ |
| neoxanthin | Neoxan | 10.3, 583.4144 | ChromaDex |
| ursolic/oleanic acid | Urs | 9.4, 439.3575 | Sigma-Aldrich |
| violaxanthin | Violaxan | 10.9, 601.4241 | Sigma-Aldrich |
|  |  |  |  |

a. Purification and identification were performed as described in Rudell et al. 2005.

b. Fluka Chemical Co., Milwaukee, WI.

c. Synthesized according to procedure detailed in Rudell et al. 2011.

d. ChromaDex, Inc., Irvine, CA.

e. Sigma-Aldrich Co. LLC, St. Louis, MO.

f. Co-elution with authentic standard on LC-QTOF-MS/MS

g. Standard acquired for *p*-coumaryl stearate and partial identification of other *p*-coumaryl esters from Bruce D. Whitaker. See Whitaker, B.D. 2001. J. Agric. Food Chem. 49:3787-3792h. see Protocol S1.
