# Supplementary material for: Gene expression and metabolism preceding soft scald, a chilling injury of ‘Honeycrisp’ apple fruit
Source: BMC Genomics. 2016 Oct 12;17:798. doi: 10.1186/s12864-016-3019-1 (PMC5062943; doi:10.1186/s12864-016-3019-1)
Supplement: Additional file 7: Protocol S1. — Farnesyl oleate, farnesyl linolenate, and farnesyl linoleate standard synthesis. (DOCX 14 kb) [file 12864_2016_3019_MOESM7_ESM.docx]

Protocol S1. Farnesyl oleate, farnesyl linolenate, and farnesyl linoleate standard synthesis.

10 μL farnesol (*l*) was placed into a 2 ml vacuum vial. Then 24 µl acyl chloride (oleic chloride, linolenic chloride, or linoleic chloride), 10 µl pyridine, and 1 ml of xylene were added to the farnesol. The vial was then vacuum sealed, and heated for 1 h at 200°C.
