# Supplementary material for: Gene expression and metabolism preceding soft scald, a chilling injury of ‘Honeycrisp’ apple fruit
Source: BMC Genomics. 2016 Oct 12;17:798. doi: 10.1186/s12864-016-3019-1 (PMC5062943; doi:10.1186/s12864-016-3019-1)

Figure S5. Expression levels (RPKM) of *Malus domestica* housekeeping genes from Bowen et al. (2015) in RNA-seq analyses. Vertical bars represent standard error of the mean (n=3).


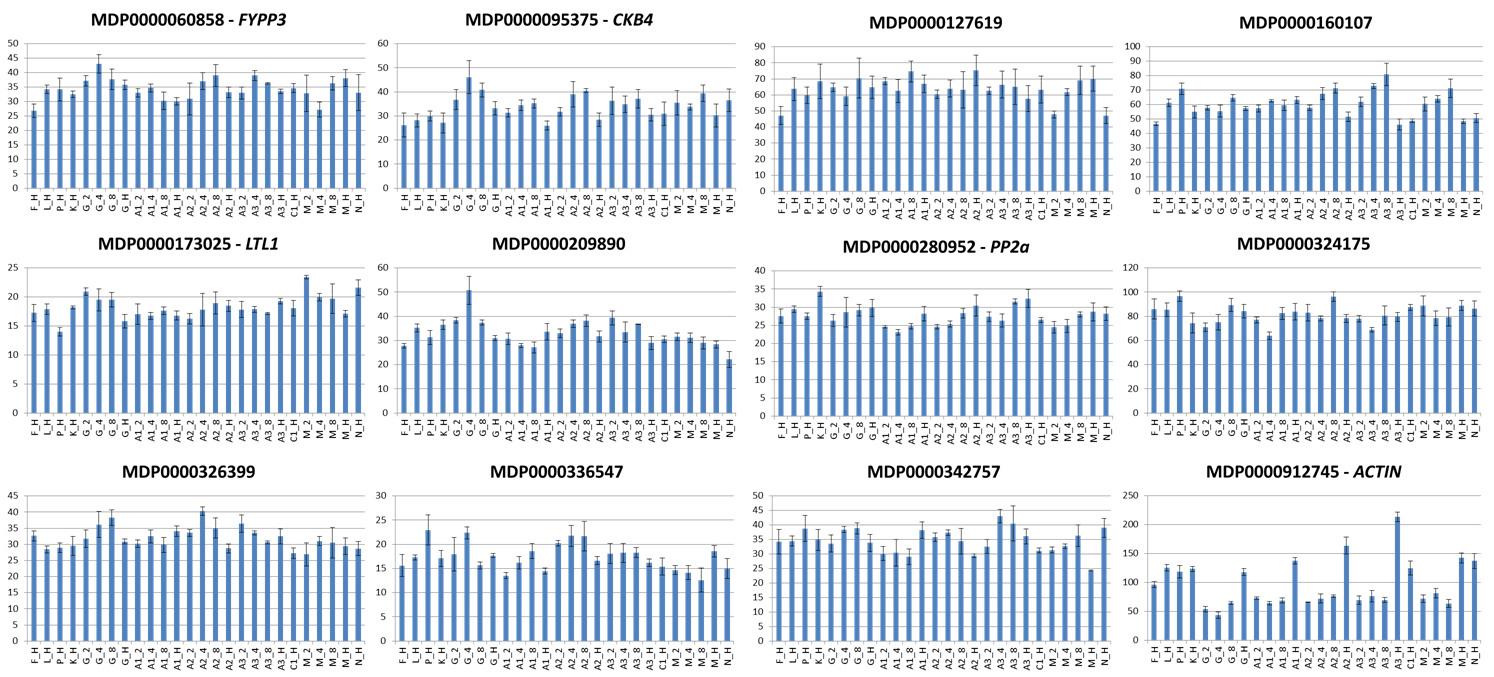

Supplement: Additional file 8: Figure S5. — Expression levels (RPKM) of Malus domestica housekeeping genes from Bowen et al. (2015) in RNA-seq analyses. (DOCX 174 kb) [file 12864_2016_3019_MOESM8_ESM.docx]
